# An Innovative Aggregation-Induced Emission-Based NIR Fluorescent Probe for Visualizing Carboxylesterases in Living Cells, Zebrafish, and Tumor-Bearing Mice

**DOI:** 10.3390/molecules29153660

**Published:** 2024-08-02

**Authors:** Chao Gao, Dan-Dan Chen, Hu-Wei Liu, Ming-Lan Ma, Lin Zhang, Hai-Rong Cui

**Affiliations:** Synergy Innovation Centre of Biological Peptide Antidiabetics of Hubei Province, College of Life Science, Wuchang University of Technology, Wuhan 430223, China; 120160880@wut.edu.cn (C.G.); dd262029@foxmail.com (D.-D.C.); hwliu@pku.edu.cn (H.-W.L.); maminglan8282@foxmail.com (M.-L.M.); 120160221@wut.edu.cn (L.Z.)

**Keywords:** aggregation-induced emission, NIR fluorescent probe, carboxylesterases, bioimaging

## Abstract

In the human body, carboxylesterases (CEs) play crucial roles in xenobiotic metabolism and lipid homeostasis. But abnormal expression of CEs is highly associated with some diseases, such as hyperlipidemia, diabetes, and liver cancer. Therefore, it is of great importance to develop an efficient tool for the accurate detection of CEs in living organisms. Herein, an innovative near-infrared (NIR) fluorescent probe, **TTAP**−**AB,** was designed for CE detection based on the aggregation-induced emission (AIE) mechanism. This probe exhibits rapid response (2 min), excellent sensitivity (limit of detection = 8.14 × 10^−6^ U/mL), and high selectivity to CEs. Additionally, owing to its good biocompatibility, the **TTAP**−**AB** probe enables the monitoring of dynamic changes in CE levels under drug-induced modulation in living cells and zebrafish. More importantly, the **TTAP**−**AB** probe was successfully employed to image liver tumors and assist in tumor resection through the real-time monitoring of CEs, indicating that **TTAP**−**AB** is promising to guide liver cancer surgery. Therefore, the **TTAP**−**AB** probe can not only enrich the strategies for CE detection in biological systems but also has great potential for some clinical imaging applications, including medical diagnosis, preclinical research, and imaging-guided surgery.

## 1. Introduction

Carboxylesterases (CEs, with EC 3.1.1.1), as essential members of the serine hydrolase superfamily, are found in various tissues of the human body, especially the liver and intestine [1]. CEs play key roles in catalyzing the hydrolysis of endogenous esters, thioesters, carbonates, carbamates, and amides. In addition, they are involved in the metabolic elimination of various xenobiotics, including ester prodrugs, pesticides, and environmental toxicants [2]. Apart from metabolizing various exogenous and endogenous substances, CEs also have vital physiological functions in lipid homeostasis that convert monoacylglycerides into free fatty acids [3]. However, abnormal CE expression is tightly correlated with many diseases, such as hyperlipidemia, diabetes, atherosclerosis, and even liver cancer [4,5]. Therefore, exploring an effective strategy to track the distribution of CEs and evaluate their activity variation in cells or tissues is of great significance for both clinical diagnosis and the treatment of various diseases.

There have already been various methods developed for the detection of CEs, including mass spectrometry [6], chromatography [7], chemiluminescence [8], and the fluorescent probe [9]. Among them, the fluorescent probe has attracted much attention due to its advantages of simple operation, fast detection, high spatiotemporal resolution, and noninvasive imaging in living systems [9]. At present, plenty of fluorescent probes have been reported based on various mechanisms, including photoinduced electron transfer (PET) [10,11,12], intramolecular charge transfer (ICT) [13,14,15,16,17,18], and other mechanisms [19,20,21,22]. However, these probes still suffer from various drawbacks in practical applications. First, most of them required a long time (≥10 min) to accomplish the fluorescence response [10,11,12,13,14,15,17,18,19,20,21,22]; thus, they were unsuitable for the real-time monitoring of CEs in living organisms. In addition, some probes were severely hydrophobic, and they required a large volume fraction of toxic organic cosolvents [11,13,15,17], which was harmful for their applications in the analysis of real biological samples. Even worse, many probes outputted the fluorescence signals in the visible region (<650 nm) [10,11,12,17,20,21,22], which might result in low tissue penetration ability and serious tissue damage. By contrast, near-infrared (NIR) fluorescent probes (650–900 nm) were more suitable for biological testing in vivo because they exhibited low background interference, excellent tissue penetrability, and low tissue damage [23,24]. The characteristics of the above fluorescent probes are summarized in Table S1. Therefore, there is an urgent need to develop a water-soluble NIR fluorescent probe for the rapid, sensitive, and selective detection of CEs in biological systems.

Aggregation-induced emission (AIE), a unique optical phenomenon whereby a class of luminogens were nearly non-emissive in a dilute solution but highly emissive in the aggregated state, was first coined by Tang et al. in 2001 [25]. In this regard, turn-on bioprobes could be easily constructed by taking advantage of the AIE effect. It was believed that the nanoaggregates of the AIE-based probes would possess better photostability and higher signal reliability than the single molecule of conventional probes. Furthermore, the negligible background noise of AIE-based probes rendered them especially attractive for the continuous monitoring of biological processes without repeated washing steps [26]. On account of the above advantages, AIE-based probes have been widely used in bioimaging and biological testing [27]. But, as far as we know, the AIE-based fluorescent probes for CE detection have been rarely reported. Dai and co-workers reported an AIE-based fluorescent probe for monitoring CEs in HepG2 cells, but the fluorescence signal was in the visible region (λ_em_ = 589 nm) [22]; therefore, this probe was unsuitable for the fluorescence detection of CEs in vivo.

**Scheme 1:**
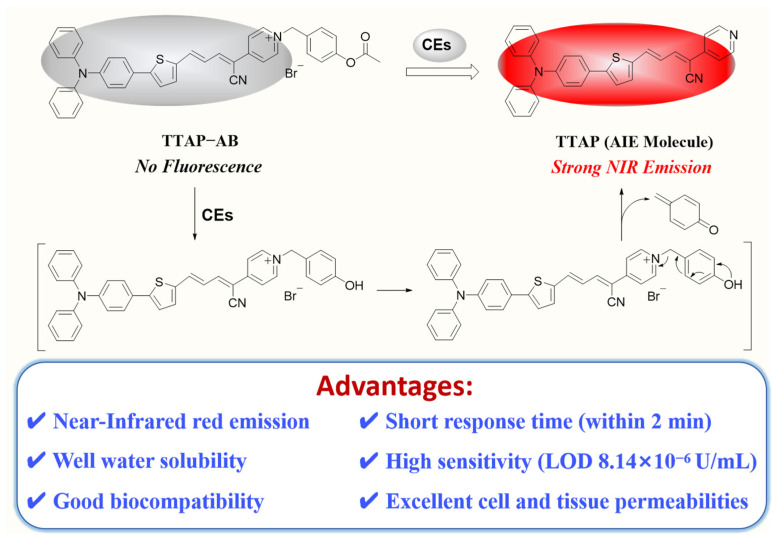
The proposed mechanism of the **TTAP**−**AB** probe to detect CEs.

Herein, we developed a novel NIR fluorescence **TTAP**−**AB** probe for detecting CEs with the AIE mechanism (Scheme 1). The **TTAP**−**AB** probe was designed based on a typical D–π–A structure, in which the conjugated C=C double bond bridged the triphenylamine–thiophene (**TT**) skeleton (electron donor, D) and the acrylonitrile–pyridinium (**AP**) moiety (electron acceptor, A). Owing to the cationic pyridinium moiety, the **TTAP**−**AB** probe was well dissolved in the PBS buffer. As the presence of CEs increased, the acetoxy–benzyl recognition group was hydrolyzed and broken by CEs, which triggered the self-elimination reaction and finally released the AIE-active fluorophore **TTAP**. Because **TTAP** was not dissolved in the PBS buffer, its aggregation aroused an intense NIR emission (λ_em_ = 692 nm). In addition, the **TTAP**−**AB** probe exhibited a high selectivity, fast response (within 2 min), and low limit of detection (LOD: 8.14 × 10^−6^ U/mL) for CEs. Owing to its good biocompatibility and excellent cell–tissue penetrability, **TTAP**−**AB** could monitor the dynamic change in CE levels induced by 5-fluorouracil (an anti-tumor drug) and CE inhibitors in living cells and zebrafish. More importantly, **TTAP**−**AB** was successfully employed to image the liver tumor and assist tumor resection through the real-time monitoring of CEs, indicating that the **TTAP**−**AB** probe was promising to guide liver cancer surgery.

## 2. Results and Discussion

### 2.1. Synthesis and Characterization of the **TTAP−AB** Probe

**Scheme 2:**
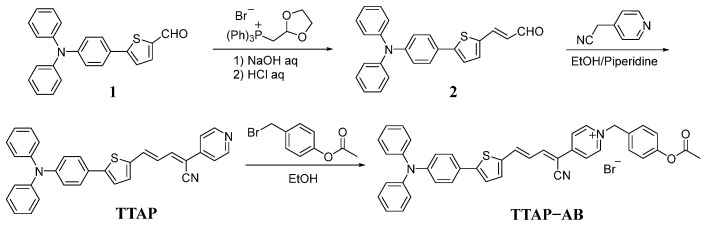
The synthetic route of the **TTAP**−**AB** probe.

As shown in Scheme 2, the **TTAP**−**AB** probe was synthesized via a three-step reaction. Firstly, the Wittig reaction of compound **1** and (1,3-dioxolan-2-ylmethyl)triphenylphosphonium bromide produced intermediate **2**. Subsequently, a Knoevenagel condensation reaction was performed using compound **2** and 2-(pyridin-4-yl)acetonitrile to produce fluorophore **TTAP**. Finally, the subsequent quaternization reaction of **TTAP** with 4-(bromomethyl)phenyl acetate produced the **TTAP**−**AB** probe with a 62% yield. The structures of the above compounds were confirmed using ^1^H NMR, ^13^C NMR, and HRMS, as shown in the Supporting Information (Figures S8–S15).

### 2.2. Spectroscopic Response of **TTAP−AB** to CEs

**Figure 1:**
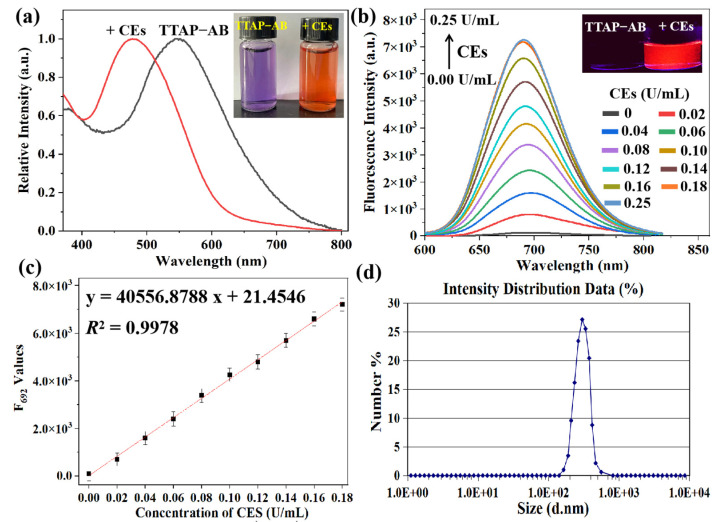
(**a**) Normalized absorption of the **TTAP**−**AB** probe (5 μM) with respect to 0.25 U/mL CEs. Inset: the corresponding photographs taken under sunlight. (**b**) Fluorescence spectra of **TTAP**−**AB** (5 μM) with respect to CEs (0.00–0.25 U/mL). λ_ex_ = 505 nm; slit width: 5 nm. Inset: the corresponding photographs taken under 365 nm light. (**c**) The fitted linear relationship of fluorescence intensity at 692 nm versus CE concentrations. (**d**) DLS profiles of **TTAP**−**AB** (5 μM) with 0.25 U/mL of CEs in PBS buffer. Error bars: standard deviation (SD), experimental times: *n* = 3.

First, the absorption and fluorescence spectra of the pure **TTAP**−**AB** probe were investigated in the absence and presence of CEs; as shown in Figure 1a, **TTAP**−**AB** (5 μM) showed an absorption peak at 548 nm and negligible fluorescence in the PBS buffer. Upon treatment with CEs, a new absorption peak was observed at 480 nm, accompanied by a change in color from purple to orange, which may have been caused by a hydrolytic reaction. According to the formula “A = εbc”, the absorption coefficients of **TTAP**−**AB** before and after treatment with CEs were calculated to be 4.17 × 10^4^ L·mol^−1^·cm^−1^ and 1.10 × 10^5^ L·mol^−1^·cm^−1^, respectively (Figure S1). Meanwhile, the fluorescence intensity at 692 nm (F_692_) was enhanced continuously upon a gradual increase in CE concentration (0.00–0.25 U/mL) (Figure 1b). Notably, the F_692_ values exhibited a good linear relationship (*R*^2^ = 0.9978), with CE concentrations ranging from 0.00 to 0.18 U/mL (Figure 1c). Based on the formula 3σ/k, the limit of detection (LOD) was calculated to be 8.14 × 10^−6^ U/mL (Section S2), implying a much higher sensitivity than many other CE probes [10,11,13,14,15,19,21,22]. Moreover, as shown by the dynamic light scattering (DLS) data, an initial signal peak was not found, indicating that the **TTAP**−**AB** probe dissolved and dispersed well in the PBS buffer. However, after treatment with 0.25 U/mL of CEs, the average size of the formed nanoparticles was around 240 nm (Figure 1d). It is clear that the CEs caused the aqueous **TTAP**−**AB** solution to form nanoaggregates, consequently resulting in bright NIR emissions.

### 2.3. The Studies of Selectivity, Response Time, and pH Effect

**Figure 2:**
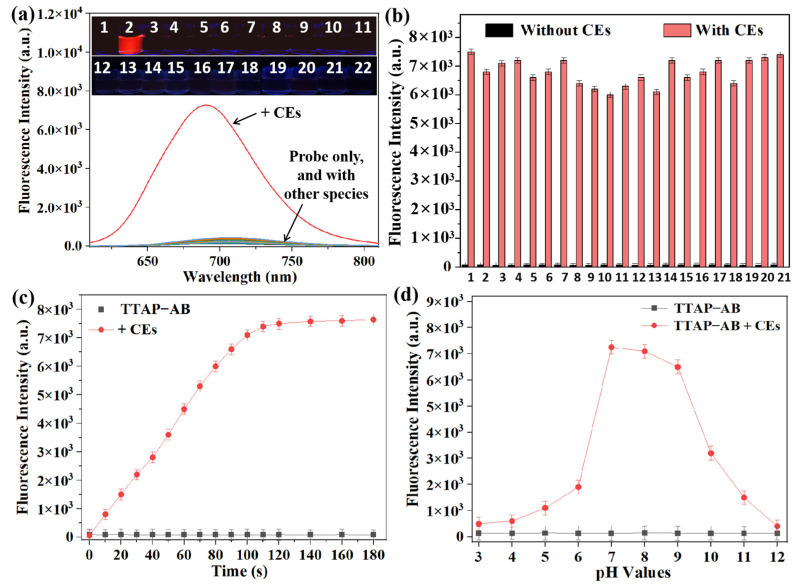
(**a**) Fluorescence spectra of the **TTAP**−**AB** probe (5 μM) in response to various species (0.20 U/mL CEs, 0.60 U/mL other enzymes, 100 μM different amino acids, and ions) in a PBS buffer (10 mM, pH 7.4). 1: blank; 2: CEs; 3: AchE; 4: CAI; 5: XO; 6: POD; 7: CPA; 8: LAP; 9: Glu; 10: Cys; 11: GSH; 12: Hcy; 13: K^+^; 14: Na^+^; 15: Zn^2+^; 16: Cu^2+^; 17: Mg^2+^; 18: Cl^−^; 19: CO_3_^2−^; 20: SO_3_^2−^; 21: S^2−^; 22: H_2_PO_4_^−^. Insert: The corresponding photos taken under 365 nm light. (**b**) The fluorescence intensity of **TTAP**−**AB** (5 μM) in the presence of various analytes (100 μM or 0.60 U/mL) without and with CEs (0.20 U/mL). 1: blank; 2: AchE; 3: CAI; 4: XO; 5: POD; 6: CPA; 7: LAP; 8: Glu; 9: Cys; 10: GSH; 11: Hcy; 12: K^+^; 13: Na^+^; 14: Zn^2+^; 15: Cu^2+^; 16: Mg^2+^; 17: Cl^−^; 18: CO_3_^2−^; 19: SO_3_^2−^; 20: S^2−^; 21: H_2_PO_4_^−^. (**c**) The time-dependent experiments of **TTAP**−**AB** (5 µM) without and with CEs (0.20 U/mL). (**d**) Fluorescence intensity changes of **TTAP**−**AB** (5 µM) in the presence of CEs (0.20) under different pH conditions. The fluorescence intensity was recorded at 692 nm. λ_ex_ = 505 nm. Error bars: standard deviation (SD), experimental times: *n* = 3.

To evaluate its selectivity toward CEs, the **TTAP**−**AB** probe was incubated with various potential interferents, including 0.60 U/mL of a common enzyme (acetyl cholinesterase (AchE), carbonic anhydrase I (CAI), xanthine oxidase (XO), peroxidase (POD), carboxypeptidase A (CPA), and leucine aminopeptidase (LAP)); 100 μM each of amino acids (glutamic acid (Glu), cysteine (Cys), glutathione (GSH), and homocysteine (Hcy)); and 100 μM each of common ions (K^+^, Na^+^, Zn^2+^, Cu^2+^, Mg^2+^, Cl^−^, CO_3_^2−^, SO_3_^2−^, S^2−^, and H_2_PO_4_^−^). The fluorescence of **TTAP**−**AB** at 692 nm was only marginally triggered by CEs (Figure 2a), and the quantum yield of **TTAP**−**AB**+**CEs** (Φ = 0.384) was almost 15-fold higher than that of **TTAP**−**AB** (Φ = 0.025) (Section S3); conversely, the other species caused negligible fluorescence changes. To further investigate the CEs-dependent selective response, an anti-interference test was carried out. As shown in Figure 2b, even when coexisting with different interferents, CEs could still significantly enhance the fluorescence intensity of **TTAP**−**AB**. The carboxyl groups of Cys and GSH led to the protonation of pyridine moieties in **TTAP**, which improved the water solubility of **TTAP** and reduced its aggregation in the PBS buffer, consequently causing a slight decrease in fluorescence intensity (Figure 2b). In summary, these results suggest that the **TTAP**−**AB** probe possesses a relatively high selectivity for CEs compared to other interferential species, setting a solid foundation for CE detection in subsequent biological experiments.

Previous reports show that most fluorescent probes require a long response time (over 10 min) for CEs [10,11,12,13,14,15,17,18,19,20,21,22], making them unsuitable for real-time analysis in living organisms. In view of this, we studied the time-dependent fluorescence intensity response of the **TTAP**−**AB** probe to CEs treatment. When **TTAP**−**AB** (5 μM) was excited at 505 nm, its fluorescence intensity remained unchanged for 30 min, indicating that it possessed excellent photostability. By contrast, when **TTAP**−**AB** was treated with CEs (0.20 U/mL), the fluorescence intensity at 692 nm increased rapidly and reached a plateau within 2 min (Figure 2c). It is clear that **TTAP**−**AB** exhibited a much faster response to CEs than most reported probes (Table S1). For broader biological applications, we investigated the impacts of environmental pH on the fluorescence response of the **TTAP**−**AB** probe when treated with CEs. As displayed in Figure 2d, the fluorescence intensity of **TTAP**−**AB** (5 μM) was basically not affected at different pHs, indicating that **TTAP**−**AB** displayed excellent pH stability. After treatment with CEs (0.20 U/mL), the fluorescence intensity at 692 nm was significantly increased when the pH was 6.0–9.0. In a strongly acidic medium (pH ≤ 5), the **TTAP** produced from **TTAP**−**AB** treated with CEs was dissolved in an aqueous solution due to protonation and thus was unable to emit in the NIR, while in strong alkaline conditions (pH ≥ 10), the reaction of CEs toward **TTAP**−**AB** was reduced [15,18], which hampered the release of fluorophore **TTAP** and prevented the generation of NIR fluorescence. Despite this, the **TTAP**−**AB** probe was still able to monitor CEs in physiological environments.

### 2.4. Sensing Mechanism

**Figure 3:**
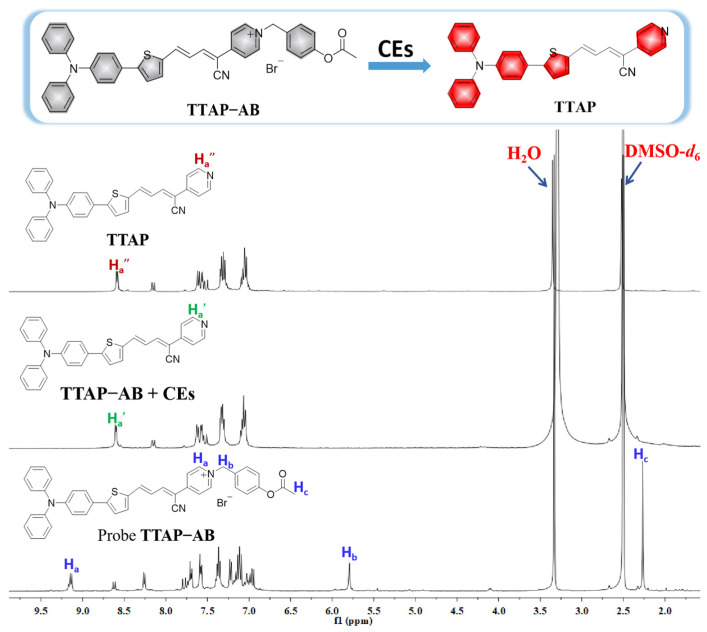
^1^H NMR spectra of compound **TTAP**, the **TTAP**−**AB** probe, and the isolated product of **TTAP**−**AB** with CEs conducted in DMSO-*d*_6_.

To confirm the reaction mechanism, the product of the reaction between **TTAP**−**AB** and CEs was separated and purified (Section S4), and its structure was analyzed using HRMS and ^1^H NMR. As shown in Figure S2, the HRMS spectrum displayed a major peak at *m*/*z* = 482.16848 [M + H]^+^, indicating that the **TTAP**−**AB** probe was hydrolyzed by CEs to release the fluorophore **TTAP**. Moreover, according to the ^1^H NMR spectra of **TTAP**−**AB**, **TTAP**, and the purified **TTAP**−**AB**+**CEs** product (Figure 3), the signals of the protons in the **TTAP**−**AB** acetoxy-benzyl recognition group at 5.79 ppm (H_b_) and 2.27 ppm (H_c_) both disappeared after reacting with CEs. Meanwhile, the signals of protons on the pyridine ring (H_a_) in **TTAP**−**AB** showed a distinct upfield shift from 9.14 ppm to 8.15 ppm after the reaction. More importantly, the ^1^H NMR spectra of the product from **TTAP**−**AB**+**CEs** were almost the same as those of **TTAP**. The above results demonstrate that the reaction of **TTAP**−**AB** with CEs produced the fluorophore **TTAP**.

**Figure 4:**
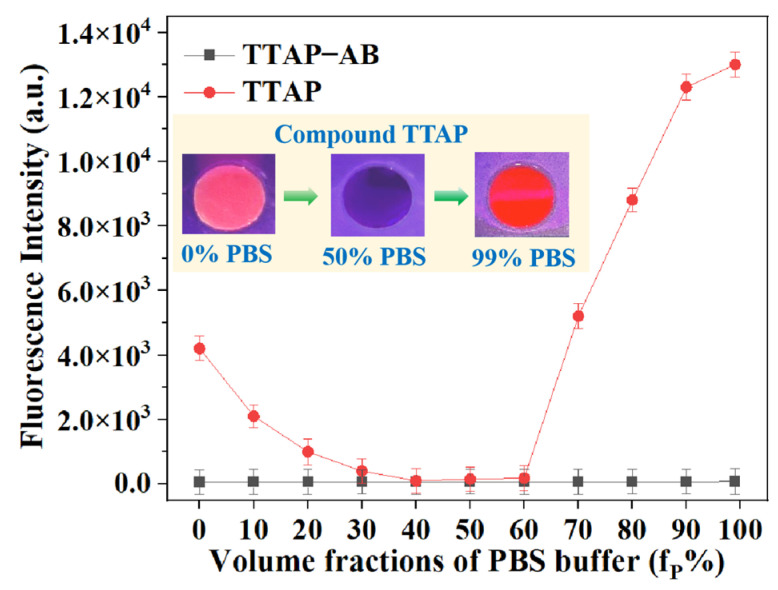
The fluorescence intensity of the **TTAP**−**AB** probe (15 μM) and compound **TTAP** (15 μM) in DMSO–PBS buffer mixture with different volume fractions of PBS buffer (f_P_ %). Insert: the photos of **TTAP** in different solutions taken under 365 nm light. Error bars: standard deviation (SD), experimental times: *n* = 3.

For a clearer understanding of the “turn-on” fluorescence response of the **TTAP**−**AB** probe to CEs, the fluorescence spectra of **TTAP**−**AB** and **TTAP** were studied in a DMSO–PBS mixture with different volume fractions of PBS buffer (f_P_). As shown in Figure 4, **TTAP**−**AB** was almost non-emissive in pure DMSO, and the continuous addition of PBS buffer caused a negligible impact on fluorescence intensity. When f_P_ reached levels of up to 99%, **TTAP**−**AB** formed nanoaggregates with an average size of 124 nm in the solution (Figure S3a), but the fluorescence intensity was hardly enhanced. Therefore, the **TTAP**−**AB** probe was shown to be non-emissive in both a dilute solution and an aggregate state, which might have been caused by the strong intramolecular charge transfer (ICT) effect from the triphenylamine–thiophene skeleton (strong electron donor) to the acrylonitrile–pyridinium moiety (strong electron donor), as well as the intense intermolecular dipole−dipole interaction [28,29]. Meanwhile, the **TTAP** compound exhibited a weak pink emission in pure DMSO. Upon an increase in f_P_, the fluorescence intensity was slightly decreased because the PBS buffer improved the solvent polarity, which compelled the **TTAP** compound to form a twisted charge-separated conformation via intramolecular rotation, consequently weakening the fluorescence intensity [30]. When f_P_ reached levels of up to 70%, the fluorescence intensity at 692 nm was dramatically improved; when f_P_ = 99%, the fluorescence intensity reached the maximum possible level, and the DLS results revealed that this solution formed nanoaggregates with an average size of 357 nm (Figure S3b). Therefore, the **TTAP** compound possessed AIE activity, and its aggregation was responsible for the **TTAP**–**AB** probe’s NIR fluorescence response toward CEs.

### 2.5. Cell Imaging of CEs

**Figure 5:**
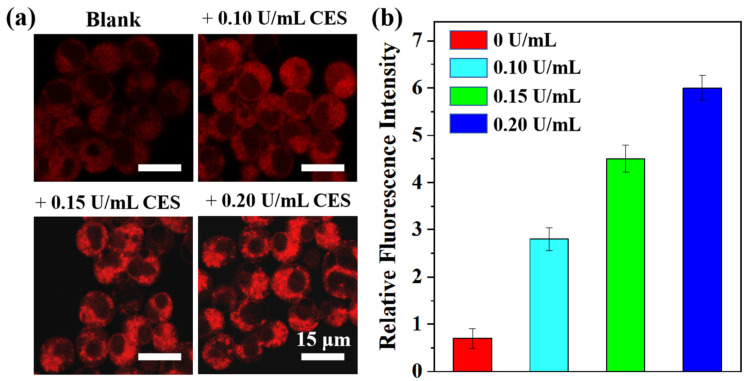
(**a**) Confocal imaging of the different concentrations of CEs in living HepG2 cells; λ_ex_ = 488 nm; λ_em_ = 650–750 nm. (**b**) Relative intensities of cell imaging. Error bars: standard deviation (SD), experimental times: *n* = 3.

Inspired by the remarkable CE-sensitivity of the **TTAP**−**AB** probe in vitro, its application in living cells was then investigated. HepG2 cells were chosen as the model cell line due to their high CE expression [12]. Prior to cell imaging, the cytotoxicity of **TTAP**−**AB** was evaluated using standard MTT assays. As shown in Figure S4, even if the concentration of **TTAP**−**AB** co-cultured with cells reached 25 μM, the cell survival rate was over 90%, indicating **TTAP**−**AB**’s low cytotoxicity toward HepG2 cells and its suitability for the subsequent imaging experiments. Afterward, the sensing behavior of the **TTAP**−**AB** probe toward CEs in HepG2 cells was investigated. When the HepG2 cells were stained with only 5 μM **TTAP**−**AB** for 30 min, weak intracellular NIR fluorescence was observed (Figure 5a); when the cells were further incubated with different concentrations of CEs (0.10 U/mL, 0.15 U/mL, and 0.20 U/mL) for another 1 h, the emission intensities in the NIR channel showed a steady improvement with an increase in probe concentration (Figure 5b). Notably, the relative fluorescence intensity increased linearly with CEs levels from 0 to 0.20 U/mL (Figure S5), suggesting that **TTAP**−**AB** has promise for quantifying the levels of CEs in living cells. Thus, the **TTAP**−**AB** probe was able to image exogenous CEs in living cells.

**Figure 6:**
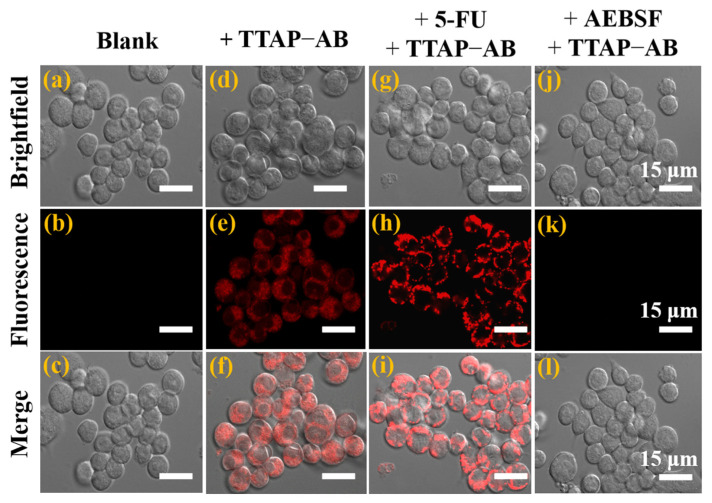
Confocal imaging of CEs in living HepG2 cells. (**a**–**c**) Untreated cells. (**d**–**f**) Cells incubated with the **TTAP**−**AB** probe (5 μM) for 1 h. (**g**–**i**) Cells successively treated with 5-FU (100 μM) for 6 h and **TTAP**−**AB** (5 μM) for 1 h. (**j**–**l**) Cells successively treated with AEBSF (1 mM) for 2 h and **TTAP**−**AB** (5 μM) for another 1 h. Incubation temperature: 37 °C; λ_ex_ = 488 nm, λ_em_ = 650–750 nm.

According to previous reports, CEs participate in the metabolism of many clinical drugs which also regulate their activity [1]. Therefore, detecting CEs activity is extremely important for studying the relationship between CEs and drug metabolism, which will provide guidance for using drugs reasonably in therapies for related diseases. 5-fluorouracil (5-FU), an anti-tumor drug, has been proven to up-regulate CEs activity in HepG2 cells [12,16]. In view of this, the feasibility of using the **TTAP**−**AB** probe to monitor the activity of CEs regulated by 5-FU was investigated. As shown in Figure 6b, the untreated HepG2 cells had no background fluorescence, while intracellular NIR fluorescence could be clearly seen after co-incubation with **TTAP**−**AB** (5 μM) for 1 h (Figure 6e). Moreover, when HepG2 cells were successively treated with 5-FU (100 μM) and **TTAP**−**AB** (5 μM), the fluorescence intensity was obviously enhanced (Figure 6h and Figure S6). However, when the cells were pre-incubated with 1 mM AEBSF (4-(2-Aminoethyl)benzenesulfonyl fluoride hydrochloride, a CEs inhibitor) for 2 h, the fluorescence intensity in the NIR channel was significantly blocked (Figure 6k). These results show that the **TTAP**−**AB** probe could monitor the dynamic changes in CEs levels induced by the drug 5-FU and CEs inhibitors in living cells.

### 2.6. Visualization of CEs Levels in Zebrafish

**Figure 7:**
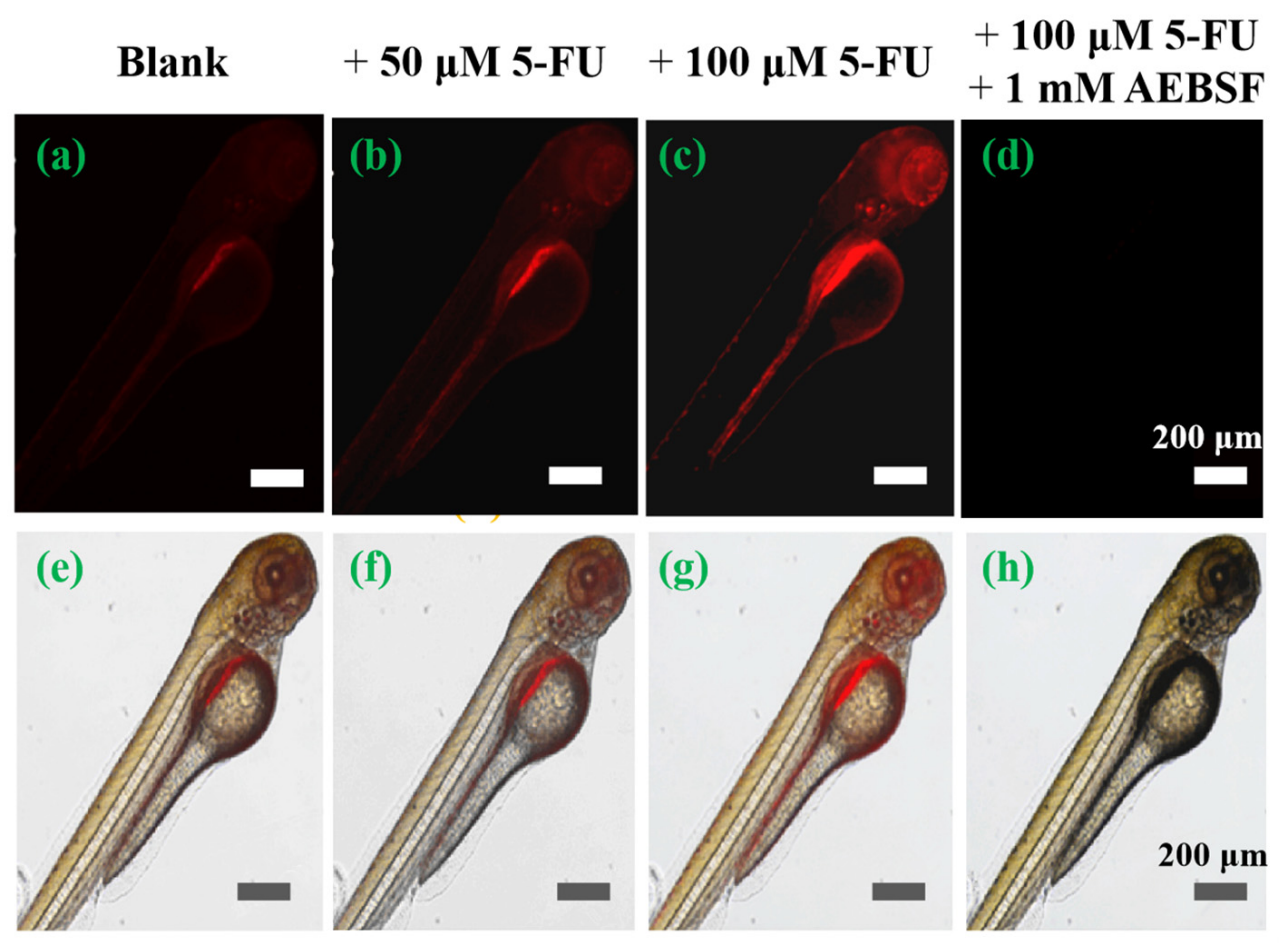
Imaging the dynamic change of CE levels in living zebrafish. Cells were stained with **TTAP**−**AB** (5 μM) only as a control (**a**,**e**), with 50 μM 5-FU (**b**,**f**), with 100 μM 5-FU(**c**,**g**), and with 100 μM 5-FU and 1 mM AEBSF (**d**,**h**). λ_ex_ = 488 nm; λ_em_ = 650–750 nm.

Encouraged by its remarkable cell imaging performance, the feasibility of using the **TTAP**−**AB** probe to visualize CEs in vivo was studied, using zebrafish larvae as a vertebrate model. As shown in Figure 7, the zebrafish exhibited weak NIR emissions in the abdomen after treatment with **TTAP**−**AB** (5 μM) only. This was mainly due to the normal levels of CEs in zebrafish. When treated with different concentrations of 5-FU (50 μM, 100 μM) for 10 h, the probe-loaded zebrafish showed bright NIR fluorescence. In addition, the intensity of NIR fluorescence notably increased with increasing 5-FU concentration (Figure 7b,c), indicating that more endogenous CEs were being generated. However, after further incubation with AEBSF, the fluorescence signals decreased rapidly (Figure 7d), mainly due to the decomposition of the CEs. These results were consistent with those of cell imaging. These results indicate that the **TTAP**−**AB** probe was able to penetrate the tissues and monitor dynamic changes in CE levels in zebrafish.

### 2.7. Bioimaging of CEs in Tumor-Bearing Mice

**Figure 8:**
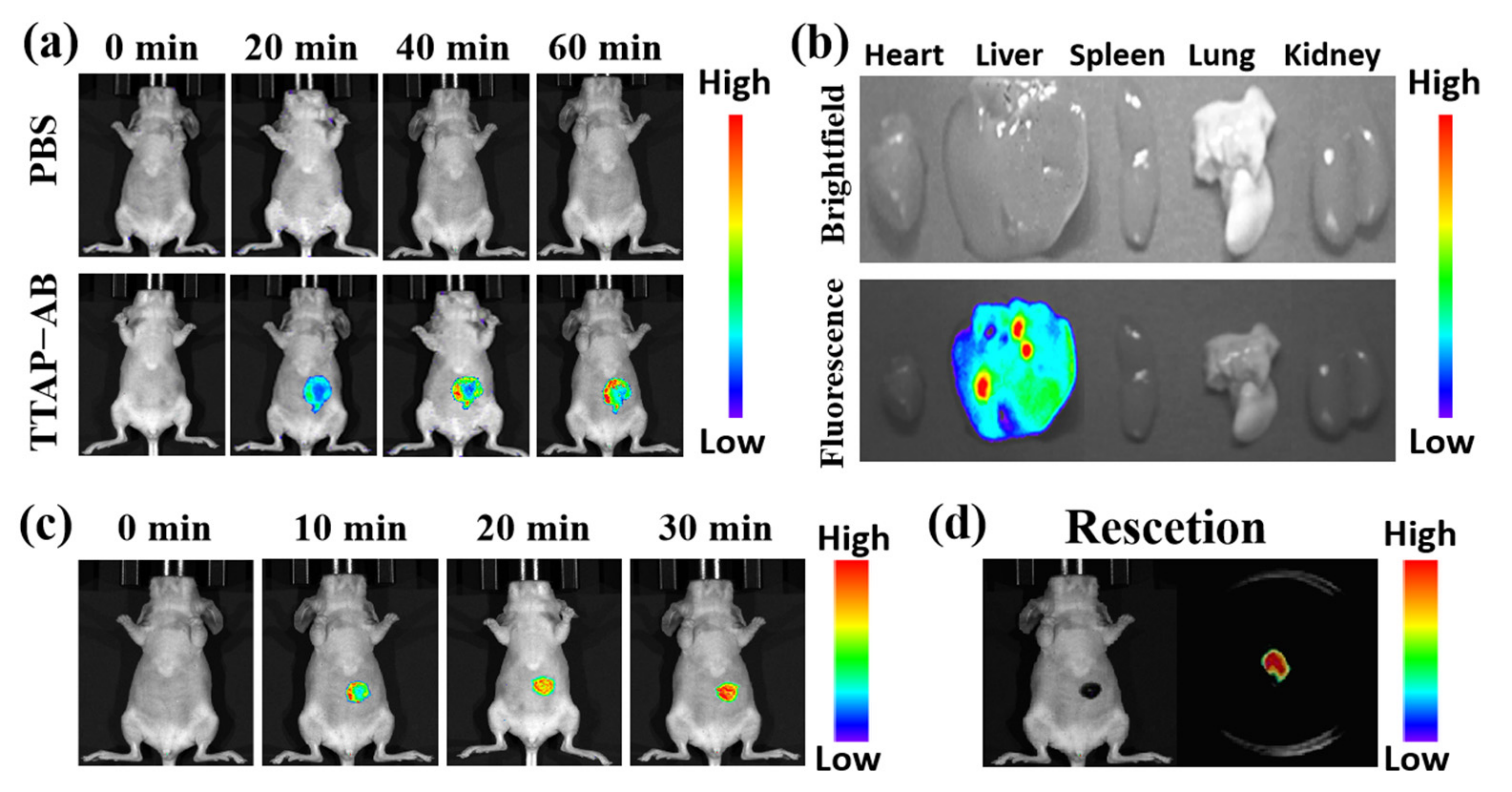
(**a**) Real-time imaging of mice injecting with PBS (100 μL) and the **TTAP**−**AB** probe (100 μL, 200 μM). (**b**) Ex vivo imaging of the major organs in mice dissected after euthanasia. (**c**) Real-time imaging of the liver tumor injecting with **TTAP**−**AB** (20 μL, 100 μM). (**d**) Ex vivo imaging of the tumor cut from the mouse body after euthanasia. λ_ex_ = 600 nm, λ_em_ = 650–750 nm.

As previously reported, CEs are mainly distributed in the liver, and their activity is closely related to cholesterol-induced liver injury and disease, especially liver cancer [12,19]. In view of this, the ability of the **TTAP**−**AB** probe to image CEs in tumor-bearing mice was investigated. Before fluorescence imaging, the bio-safety of **TTAP**−**AB** was investigated in nude mice using histologic staining (hematoxylin and eosin, H&E) assays for 24 h. As shown in Figure S7, the **TTAP**−**AB** probe did not cause significant pathological changes in several primary organs, including the heart, liver, lung, kidney, and spleen; therefore, it showed no obvious toxicity in vivo and was considered suitable for imaging CEs in mice. Next, liver imaging was carried out. The mice in the control group were only injected with saline, and no obvious fluorescence signals were observed (Figure 8a), while in the experimental group, the mice were intravenously injected with **TTAP**−**AB** (100 μL, 200 μM), and the fluorescence signals in their abdomens increased quickly and reached the maximum levels at about 60 min, indicating that **TTAP**−**AB** was hydrolytically catalyzed by CEs. Simultaneously, the biodistribution of **TTAP**−**AB** was determined. The mice were euthanized, and their organs (heart, liver, spleen, lung, and kidney) were dissected for fluorescence imaging. As shown in Figure 8b, bright fluorescence signals were clearly observed in the liver, while there was almost no fluorescence in the other organs. These results indicated that CEs were mainly distributed in the liver, and the **TTAP**−**AB** probe was able to enter and accumulate in the livers of mice for imaging.

Afterward, imaging experiments were conducted on tumor-bearing mice. HepG2 cells (human hepatocarcinoma cells) were implanted into BALB/c mice to induce the formation of liver tumors. As depicted in Figure 8c, when the tumor-bearing BALB/c mice were injected with the **TTAP**−**AB** probe (20 μL, 100 μM), the NIR signals of the tumor site showed a clear increase over time and reached the maximum intensity at 30 min, mainly due to the over-expression of CEs in liver tumors, successfully visualizing liver tumors in vivo. More remarkably, when the tumors were dissected, they still presented intense NIR fluorescence, but there were no fluorescent signals in the mice (Figure 8d), implying that **TTAP**−**AB** could assist in tumor imaging and resectioning. Overall, it is clear that the **TTAP**−**AB** probe is capable of achieving real-time imaging of CEs in liver tumors, exhibiting a great deal of potential for practical clinical applications.

## 3. Materials and Methods

### 3.1. Materials and Instruments

Unless otherwise stated, all chemicals were purchased from commercial suppliers and used without further purification. Double-distilled water and chromatographic solvents were used for fluorescence tests. A Bruker AV-400 spectrometer (Billerica, MA, USA) was employed to record ^1^H NMR and ^13^C NMR spectra. High-resolution mass spectra (HRMS) were obtained with a Thermo Scientific Q Exactive type mass spectrometer (Waltham, MA, USA). Fluorescence spectra and fluorescence quantum yield were collected using a Hitachi F-4500 fluorescence spectrometer (New Life Scientific, Cridersville, OH, USA). Dynamic light scattering (DLS) experiments were investigated with a ZEN3600 Malvern particle sizer (Counterpane Inc., Washington, DC, USA). Fluorescence images of living cells and zebrafish were obtained with a Zeiss LSM 880 confocal laser scanning microscope (Oberkochen, Germany). All fluorescence imaging of living mice was performed by a BLT AniView 600 small animal optical imaging system (Xi’an, China).

### 3.2. Synthesis of Fluorophore **TTAP**

Compound **2** was synthesized according to the previous method [31], and its synthetic procedure was exhibited in the Supporting Information (Section S5). After that, compound **2** (1.14 g, 3.00 mmol) and 4-pyridineacetonitrile (0.17 g, 3.60 mmol) were dissolved in 15 mL ethanol, and then 0.6 mL piperidine was added to the solution. This mixture was reacted at 80 °C; for 8 h under a N_2_ atmosphere. After cooling to room temperature, the mixture was concentrated using rotary evaporators, and the remaining solid was purified by column chromatography (CH_2_Cl_2_/CH_3_OH as eluent, *v*/*v* = 30:1). The final product, **TTAP,** was obtained as a dark red solid (0.93 g, 64% yield). ^1^H NMR (400 MHz, DMSO-*d*_6_): *δ* (ppm) 8.65 (d, *J* = 6.0 Hz, 2H, -PyH), 8.21 (d, *J* = 7.2 Hz, 1H, thiophene H), 7.65–7.68 (m, 2H, -PyH), 7.61–7.63 (m, 2H, thiophene H and vinyl H), 7.30–7.38 (m, 8H, -ArH and vinyl H), 7.04–7.15 (m, 8H, -ArH); ^13^C NMR (100 MHz, DMSO-*d*_6_): *δ* (ppm) 150.48, 150.13, 147.76, 147.00, 146.58, 145.77, 138.65, 137.23, 133.66, 129.74, 129.65, 126.89, 124.75, 123.87, 123.24, 122.18, 119.20, 116.14. HRMS (ESI^+^): calcd for C_32_H_23_N_3_S [M + H]^+^ 482.16854, found 482.16852.

### 3.3. Synthesis of the **TTAP−AB** Probe

Compound **TTAP** (0.72 g, 1.50 mmol) and 4-(bromomethyl)phenyl acetate (0.36 g, 1.60 mmol) were dissolved in 6 mL dry ethanol, and the mixture was then refluxed in a N_2_ atmosphere overnight. After cooling to room temperature, the mixture was concentrated using rotary evaporators, and the obtained residue was then purified using a neutral aluminum oxide column (CH_2_Cl_2_/CH_3_OH as eluent, *v*/*v* = 6:1). The final product, **TTAP**−**AB,** was obtained as a purple-black solid (0.66 g, 62% yield). ^1^H NMR (400 MHz, DMSO-*d*_6_): *δ* (ppm) 9.14 (d, *J* = 7.2 Hz, 2H, -PyH), 8.62 (d, *J* = 11.6 Hz, 1H, vinyl H), 8.26 (d, *J* = 6.8 Hz, 1H, thiophene H), 7.57–7.80 (m, 7H, -PyH, thiophene H and -ArH), 7.35–7.40 (m, 4H, -ArH), 6.94–7.23 (m, 12H, vinyl H and -ArH), 5.79 (s, 2H, -CH_2_), 2.27 (s, 3H, -CH_3_); ^13^C NMR (100 MHz, DMSO-*d*_6_): *δ* (ppm) 169.32, 159.96, 154.66, 150.81, 150.09, 149.07, 146.22, 144.61, 142.34, 135.63, 135.43, 134.69, 132.40, 131.55, 130.20, 129.87, 128.83, 127.70, 125.28, 124.93, 124.45, 122.79, 121.33, 116.64, 98.90, 61.67, 22.11. HRMS (ESI^+^): calcd for C_32_H_23_N_3_S [M-Br]^+^ 630.22097, found 630.22103.

### 3.4. Optical Study

Stock solutions of the **TTAP**−**AB** probe (5 μM) were prepared in a PBS buffer (10 mM, pH 7.4). Stock solutions of the cations (K^+^, Na^+^, Zn^2+^, Cu^2+^, Mg^2+^), anions (Cl^−^, CO_3_^2−^, SO_3_^2−^, S^2−^, H_2_PO_4_^−^), and amino acids (glutamic acid (Glu), cysteine (Cys), glutathione (GSH), and homocysteine (Hcy)) were prepared in deionized water (5 mM). Stock solutions of different enzymes (carboxylesterases, acetylcholinesterase (AchE), carbonic anhydrase I (CAI), xanthine oxidase (XO), peroxidase (POD), carboxypeptidase A (CPA), and leucine aminopeptidase (LAP)) were prepared in sterilized water (8 U/mL). The fluorescence spectra of the **TTAP**−**AB** probe (5 μM) with different concentrations of CEs or other interferents in PBS buffer (10 mM, pH 7.4) were recorded at 37 °C.

### 3.5. Living Cell Imaging

All HepG2 cells were purchased from Wuhan Mingde Biotechnology Co., Ltd. For the cytotoxicity assay, the cells were first incubated in Dulbecco’s modified Eagle medium containing 10% fetal bovine serum and then kept at 37 °C under 5% CO_2_ conditions for 24 h. After that, the cells were incubated with various concentrations of the **TTAP**−**AB** probe (5, 10, 15, 20, 25 μM) for 10 h. After washing with PBS, 3-(4,5-dimethylthiazol-2-yl)-2,5-diphenyltetrazolium bromide (MTT) was added, and the medium was incubated at 37 °C for 4 h. Finally, the absorbance was read at 490 nm using an ELISA reader (Thermo Scientific-Varioskan LUX, Thermo Fisher Scientific Inc., Waltham, MA, USA). The percentage of cell viability was calculated relative to control wells designated as 100% viable cells.

To image exogenous CEs, HepG2 cells were first stained with **TTAP**−**AB** (5 μM) for 30 min and then incubated with different concentrations of CEs (0.10 U/mL, 0.15 U/mL, and 0.20 U/mL) at 37 °C for 1 h. After washing with PBS, images were taken using a confocal fluorescence microscope (λ_ex_ = 488 nm; λ_em_ = 650–750 nm). For endogenous CE imaging, the HepG2 cells were divided into four groups. In the control group, HepG2 cells were only cultivated with PBS buffer for 30 min. In the second group, the cells were incubated with the **TTAP**−**AB** probe (5 μM) for 1 h. In the third group, HepG2 cells were pre-treated with the drug 5-fluorouracil (5-FU, 100 μM) for 6 h and then incubated with **TTAP**−**AB** (5 μM) for 1 h. In the final group, HepG2 cells were pre-treated with 4-(2-Aminoethyl)benzenesulfonyl fluoride hydrochloride (AEBSF, a CEs inhibitor, 1 mM) for 2 h and then incubated with **TTAP**−**AB** (5 μM) for another 1 h. Prior to imaging, the above cells were washed with PBS buffer (10 mM, pH = 7.4) three times, and fluorescence imaging was performed using a confocal fluorescence microscope (λ_ex_ = 488 nm; λ_em_ = 650–750 nm).

### 3.6. Zebrafish Imaging

All zebrafish experiments were approved by the Experimental Animal Ethics Committee of Wuchang University of Technology (Approval Code: 20240415-063) and conducted according to the guidelines for animal experiments. Zebrafish embryos were purchased from Shanghai FishBio Co., Ltd. (Shanghai, China). Larval zebrafish (4 days old) were used for imaging, and they were divided into four groups. In a control group, zebrafish were only cultured with the **TTAP**−**AB** probe (5 μM) at 37 °C for 1 h. In the second group, zebrafish were grown with the 5-FU drug (50 μM) for 10 h and then stained with **TTAP**−**AB** (5 μM) at 37 °C for another 1 h. In the third group, zebrafish were stained with the 5-FU drug (100 μM) for 10 h and then incubated with **TTAP**−**AB** (5 μM) at 37 °C for another 1 h. In the last group, zebrafish were first treated with 5-FU (100 μM) for 10 h, then cultivated with AEBSF (1 mM) for 4 h, and finally stained with **TTAP**−**AB** (5 μM) at 37 °C for another 1 h. All zebrafish were washed three times with embryo media and then transferred to a confocal fluorescence microscope for imaging (λ_ex_ = 488 nm; λ_em_ = 650–750 nm).

### 3.7. Fluorescence Imaging in Mice

All animal experiments were approved by the Experimental Animal Ethics Committee of Wuchang University of Technology (Approval Code: 20240415-064) and conducted according to the guidelines for animal experiments. All BALB/c mice (18–20 g) were purchased from Shanghai Slac Laboratory Animal Co., Ltd. (Shanghai, China) and operated on in accordance with Wuchang University of Technology guidelines.

For histology and immunohistochemical staining, all BALB/c mouse tissues were immediately fixed in 10% formaldehyde after sacrifice. The histological examination was carried out according to conventional methods [12,13,19] via hematoxylin and eosin (H&E) staining. The morphology of any observed lesions was classified and recorded according to the classification criteria.

For imaging CEs in vivo, the mice were divided into two groups. In the control group, the mice were intravenously injected with 100 μL of PBS. In the experimental group, the mice were intravenously injected with the **TTAP**−**AB** probe (100 μL, 200 μM) for real-time recording. All the mice were anesthetized, and in vivo imaging was performed; next, they were used for the biodistribution studies. These mice were euthanized, and their organs (heart, liver, spleen, lung, and kidney) were dissected for fluorescence measurements. The fluorescence images were obtained using a BLT AniView 600 small animal optical imaging system (China) (λ_ex_ = 600 nm, λ_em_ = 650–750 nm).

To visualize CEs in tumor-bearing mice, HepG2 cells (5 × 10^7^ cells) were subcutaneously injected into female BALB/c mice (18–20 g) to establish a mouse tumor model. After 20 days, the **TTAP**−**AB** probe (20 μL, 100 μM) was injected into the tumor-bearing mice. All the mice were anesthetized, and in vivo imaging was performed. Next, the liver tumors were removed from the mice after euthanasia. The fluorescence images were obtained using a BLT AniView 600 small animal optical imaging system (China) (λ_ex_ = 600 nm, λ_em_ = 650–750 nm).

## 4. Conclusions

In summary, a new NIR fluorescent probe, **TTAP**−**AB**, has been constructed for visualizing CEs in living systems. Under physiological conditions, the **TTAP**−**AB** probe can selectively, sensitively, and quickly detect CEs using an AIE mechanism. The sensing mechanism was confirmed via HRMS, ^1^H NMR, and fluorescence spectra. In addition, the **TTAP**−**AB** probe exhibited good biocompatibility as well as excellent cell and tissue permeability, and it was favorably employed to monitor dynamic changes in CEs levels under drug-induced modulation in living cells and zebrafish. More importantly, the **TTAP**−**AB** probe was able to image liver tumors and assist with the resection of these tumors through real-time detection of CEs, indicating that **TTAP**−**AB** has great potential for practical clinical applications.

## Data Availability

The data presented in this study are available on request from the corresponding author.

## Supplementary Materials

The following supporting information can be downloaded at: https://www.mdpi.com/article/10.3390/molecules29153660/s1; Section S1: Table S1. Summary of the recent single detection probes for CEs; Section S2: Calculation of the detection limit; Section S3: Quantum yield measurement; Section S4: The synthesis of product from the **TTAP**−**AB** probe with CEs; Section S5: Synthesis of compound **2**; Section S6: Reference; Figure S1: Absorption spectral of the **TTAP**−**AB** probe (5 μM) before and after addition of CEs 0.25 U/mL in PBS buffer; Figure S2: The HRMS data of **TTAP**−**AB** before and after treating with CEs; Figure S3: The DLS profiles of the **TTAP**−**AB** probe (15 μM) in PBS buffer (with 1% DMSO) (a) and compound **TTAP** (15 μM) in PBS buffer (with 1% DMSO) (b); Figure S4: Viability of HepG2 cells after the incubation with different concentrations of the **TTAP**−**AB** probe; Figure S5: The linear relation between relative fluorescence intensity of HepG2 cells with **TTAP**−**AB** and the concentration of CEs; Figure S6: Normalized fluorescence intensity for cell imaging; Figure S7: Representative histological sections (H&E staining) for main organs of the mice without and with the injection of the **TTAP**−**AB** probe (100 μL, 200 μM); Figure S8: ^1^H NMR (400 MHz, DMSO-*d*_6_) spectrum of compound **2**; Figure S9: ^13^C NMR (100 MHz, DMSO-*d*_6_) spectrum of compound **2**; Figure S10: ^1^H NMR (400 MHz, DMSO-*d*_6_) spectrum of **TTAP**; Figure S11: ^13^C NMR (100 MHz, DMSO-*d*_6_) spectrum of **TTAP**; Figure S12: ^1^H NMR (400 MHz, DMSO-*d*_6_) spectrum of **TTAP**−**AB**; Figure S13: ^13^C NMR (100 MHz, DMSO-*d*_6_) spectrum of **TTAP**−**AB**; Figure S14: HRMS spectrum of **TTAP**; Figure S15: HRMS spectrum of **TTAP**−**AB**. Reference [32] is cited in the Supplementary Materials.
