# Supplementary material for: An Innovative Aggregation-Induced Emission-Based NIR Fluorescent Probe for Visualizing Carboxylesterases in Living Cells, Zebrafish, and Tumor-Bearing Mice
Source: Molecules. 2024 Aug 2;29(15):3660. doi: 10.3390/molecules29153660 (PMC11314084; doi:10.3390/molecules29153660)
Supplement: Supplementary file 1 [file molecules-29-03660-s001.zip › molecules-3087224-supplementary.pdf]

An Innovative Aggregation-Induced Emission-Based NIR Fluorescent Probe for Visualizing Carboxylesterases in Living Cells, Zebrafish and Tumor-Bearing Mice

Chao Gao, Dan-Dan Chen, Hu-Wei Liu, Ming-Lan Ma,
Lin Zhang, and Hai-Rong Cui*

Synergy Innovation Centre of Biological Peptide Antidiabetics of Hubei Province,
College of Life Science, Wuchang University of Technology, Wuhan 430223, China.

* *Correspondence: E-mail:* chr@wut.edu.cn

S1. Table S1 Summary of the recent fluorescent probes for CEs

Probe' Structure	Sensing Mechanism	Response Time	Limit of Detection	Detection Medium	Emission Wavelength	Application	Reference
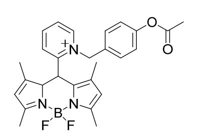	PET	10 min	3.37 × 10^{-5} U/mL	PBS buffer	517 nm	4T1 cells	**Ref. 10** *J. Org. Chem.* **2024**. **DOI**:org/10.1021/acs.joc.4c00699
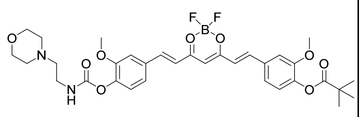	PET	30 min	3.43 × 10^{-3} U/mL	DMSO-PBS (8:2, v/v)	527 nm	HepG2 cells; HCT 116 cells**.**	**Ref. 11** *Talanta*. **2024**, *274*, 126060
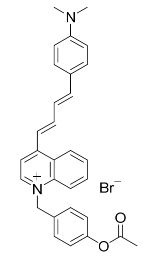	PET	40 min	3.04 × 10^{-6} U/mL	PBS buffer with 1% DMSO	593 nm	HepG2 cells; Mice.	**Ref. 12** *Anal. Chem.* **2022**, *94*, 4594−4601
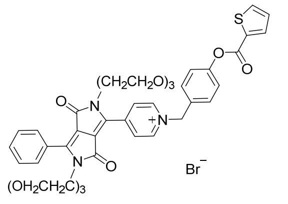	ICT	10 min	1.6 × 10^{-5} U/mL	PBS buffer with 20% EtOH	667 nm	HepG2 cells; Mice.	**Ref.13** *Talanta*. **2024**, *266*, 124971

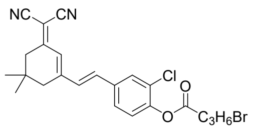	ICT	50 min	9.20 × 10^{-5} U/mL	PBS buffer	660 nm	HepG2 cells; Mice.	**Ref. 14** *Spectrochim Acta A.* **2024**, *308*, 123708
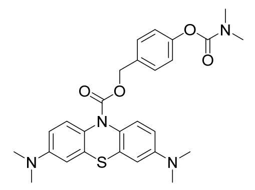	ICT	50 min	6.58 × 10^{-3} U/mL	PBS buffer with 20% DMSO	680 nm	HepG2 cells; Mice.	**Ref. 15** *Sens. Actuators B* **2024**, *402*, 135133
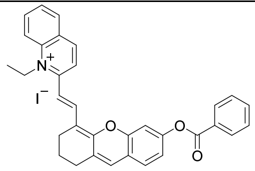	ICT	3 min	0.35 ng/mL (≈ 12.8 × 10^{-9} U/mL)	PBS buffer	765 nm	HepG2 cells; Mice.	**Ref. 16** *Sens. Actuators B* **2023**, *395*, 134503
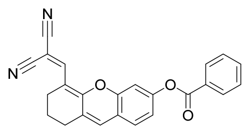	ICT	10 min	17 ng/mL (≈ 2.29 × 10^{-7} U/mL)	PBS buffer with 30% DMSO	640 nm	HepG2 cells; Zebrafish; Mice.	**Ref. 17** *Anal. Chim. Acta.* **2022**, *1221*, 340126
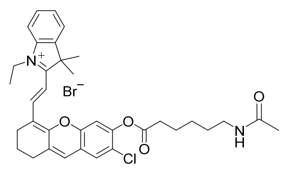	ICT	2 h	2.93 × 10^{-6} U/mL	PBS buffer with 1% DMSO	720 nm	HepG2 cells; MDA-MB-231 cells; Mice.	**Ref. 18** *Spectrochim. Acta. A* **2022**, *281*, 121529
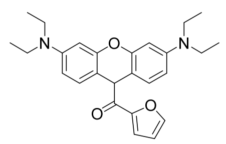	Not mentioned	60 min	1.28 × 10^{-5} U/mL	PBS buffer with 0.25% DMF	676 nm	HepG2 cells; Mice;	**Ref.19** *Adv. Sci.* **2023**, *10*, 2206681
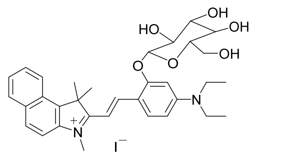	Not mentioned	30 min	6.07 × 10^{-7} U/mL	PBS buffer	595 nm	MCF-7cells; 4T1 cells.	**Ref. 20** *Chin. Chem. Lett.* **2022**, *33*, 4229–4232

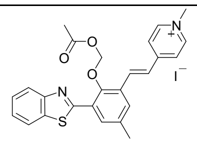	ESIPT	20 min	1.22 × 10^{-4} U/mL	PBS buffer	542 nm	HeLa cells	**Ref. 21** *Dye. Pigment.* **2021**, *191*, 109349
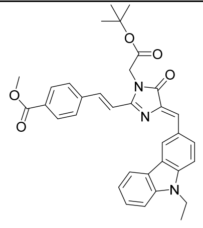	AIE	55 min	27.8 ng/mL (≈ 3.75 × 10^{-4} U/mL)	PBS buffer	589 nm	HepG2 cells	**Ref.22** *Dye. Pigment.* **2021**, *192*, 109444
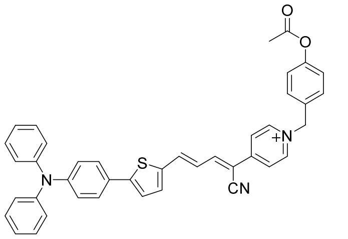	AIE	2 min	8.14 × 10^{-6} U/mL	PBS buffer	692 nm	HepG2 cells; Zebrafish; Mice.	**This work**

S2. Calculation of the detection limit

The limit of detection (LOD) of the **TTAP−AB** probe toward CEs was calculated by the following equation:

$$\text{LOD} = 3\sigma/k$$

Where σ was the standard deviation of fluorescence intensity for blank solution, which was measured for eight times. k represented the slope of the linear calibration plot between the fluorescence intensity and CEs concentration. According to the linear equation: y = 40556.8788x + 21.4546, thus LOD = 8.14×10^{-6} U/mL.

S3. Quantum yield measurement

Fluorescence quantum yields for the synthesized dyes were determined by using Indocyanine Green (ICG) (Φ_F = 0.13 in DMSO) as a fluorescence standard [S1]. The quantum yield was calculated using the following equation:

$$\Phi_{F(X)} = \Phi_{F(S)} (A_S F_X / A_X F_S) (n_X/n_S)^2$$

Where Φ_F is the fluorescence quantum yield, A is the absorbance at the excitation wavelength, F is the area under the corrected emission curve, and n is the refractive index of the solvents used. Subscripts S and X refer to the standard and to the unknown, respectively. For **TTAP−AB+CEs** and **TTAP−AB** in PBS buffer, the excitation wavelength was at 505 nm. As a result, the quantum yield (Φ) of **TTAP−AB+CEs** and **TTAP−AB** was calculated to be 0.384 and 0.025, respectively.

S4. The synthesis of product from the TTAP−AB probe with CEs

The **TTAP−AB** probe (141.8 mg, 0.2 mmol) was dissolved in dry CH_3CN (6 ml), and then 2 ml CEs aqueous solution (200 U/mL) was added into the system. Subsequently, the mixture was stirred at 30 °C for 7 h. After that, 10 ml H_2O and 15 ml CH_2Cl_2 were added into the reaction system, and the organic layer was separated. After washing with NaCl saturated

solution and H_2O for 3 times, the organic layer was dried with anhydrous Na_2SO_4, and then concentrated under vacuum. After a rapid purification using a Silicone column (DCM/CH_3OH as eluent, v/v = 30:1), the product was obtained as a brown solid (60.6 mg, 63% yield).

S5. Synthesis of compound 2

5-(4-(diphenylamino)phenyl)thiophene-2-carbaldehyde (1.42 g, 4.0 mmol) and (1,3-dioxolan-2-ylmethyl)triphenylphosphonium bromide (2.10 g, 4.9 mmol) were dissolved in dry CH_2Cl_2 (20 mL) and stirred for 5 h at room temperature. Subsequently, a solution (600 mg NaOH in 2 mL water) was added, and the resulting mixture was stirred at room temperature for 3 h. After that, 4 mL concentrated HCl was added, and the mixture was stirred for another 0.5 h. At last, the mixture was extracted with CH_2Cl_2, and the organic layer was dried over Na_2SO_4. After removing the solvent under reduced pressure, the residue was purified by a short silica gel column to afford a yellow solid (1.28 g, yield 84%). ^{1}H NMR (400 MHz, DMSO-d_6): δ (ppm) 9.59 (s, 1H, -CHO), 7.78-7.85 (m, 1H, thiophene H), 7.61-7.63 (m, 3H, -ArH and vinyl H), 7.51 (d, J = 4.0 Hz, 1H, thiophene H), 7.32-7.36 (m, 4H, -ArH), 7.06-7.11 (m, 6H, -ArH), 6.95 (d, J = 8.8 Hz, 2H, -ArH), 6.41-6.47 (m, 1H, vinyl H); ^{13}C NMR (100 MHz, DMSO-d_6): δ (ppm) 193.24, 148.49, 147.90, 146.56, 145.06, 137.00, 134.84, 129.73, 126.94, 126.20, 126.05, 124.73, 124.28, 123.87, 122.21.

S6. Reference

[S1] Oushiki, D.; Kojima, H.; Terai, T.; Arita, M.; Hanaoka, K.; Urano, Y.; Nagano, T. Development and application of a near-infrared fluorescence probe for oxidative stress based on differential reactivity of linked cyanine dyes. *J. Am. Chem. Soc.* **2010**, *132*, 2795−2801.

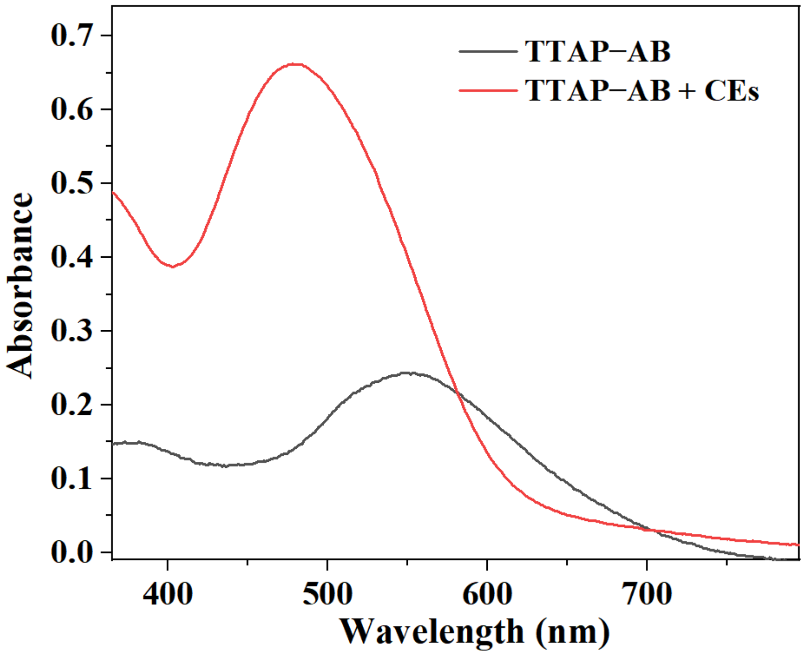

Figure S1. Absorption spectral of the **TTAP−AB** probe (5 μM) before and after addition of CEs 0.25 U/mL in PBS buffer. According to the equation "A = εbc", in which A is the absorbance (**TTAP−AB**: 0.25; **TTAP−AB+CEs**: 0.66), b is the absorption cell thickness (1.20 cm), c is the sample concentrations (**TTAP−AB**: 5 μM; **TTAP−AB+CEs**: 5 μM), thus the absorption coefficient ε before and after addition of CEs was calculated to be 4.17×10^{4} $L\cdot mol^{-1}\cdot cm^{-1}$ and 1.10×10^{5} $L\cdot mol^{-1}\cdot cm^{-1}$, respectively.

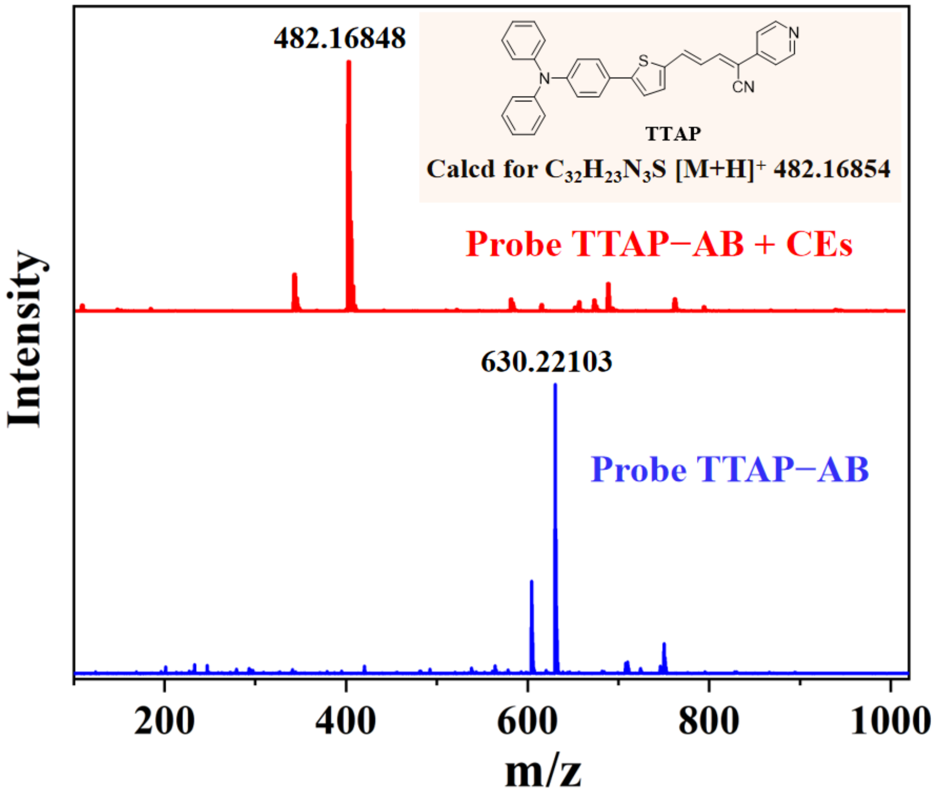

Figure S2. The HRMS data of **TTAP−AB** before and after treating with CEs

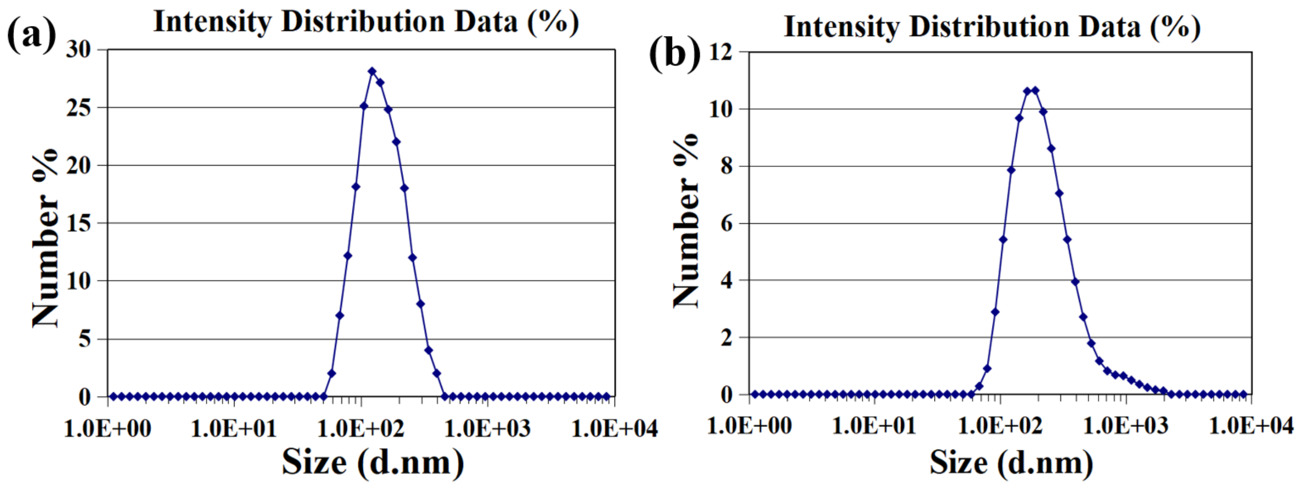

Figure S3. The DLS profiles of the **TTAP−AB** probe (15 μM) in PBS buffer (with 1% DMSO) (**a**) and compound **TTAP** (15 μM) in PBS buffer (with 1% DMSO) (**b**).

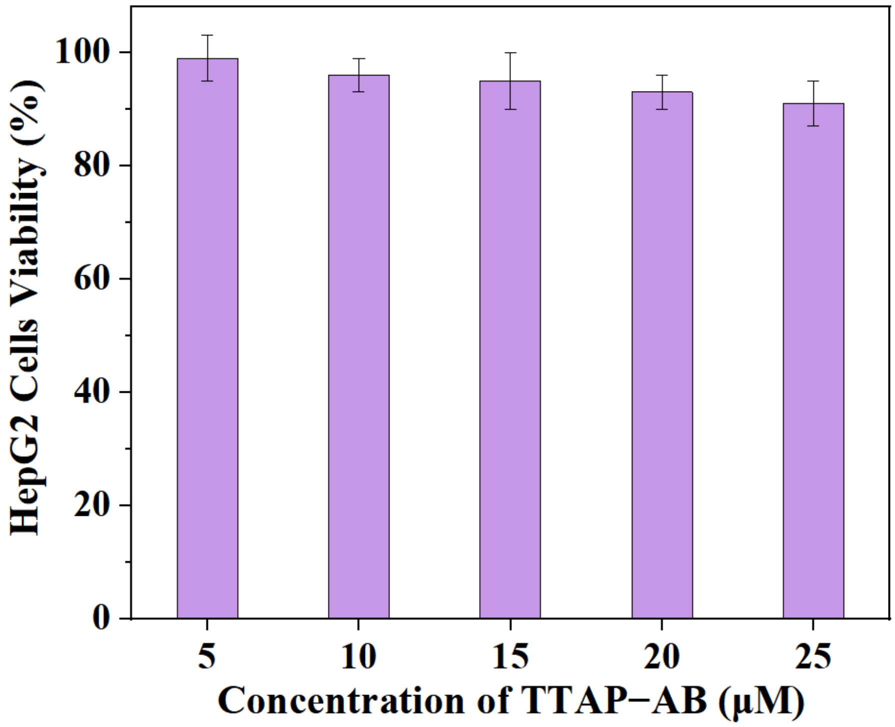

Figure S4. Viability of HepG2 cells after the incubation with different concentrations of the **TTAP−AB** probe, error bars are ± SD (n = 3).

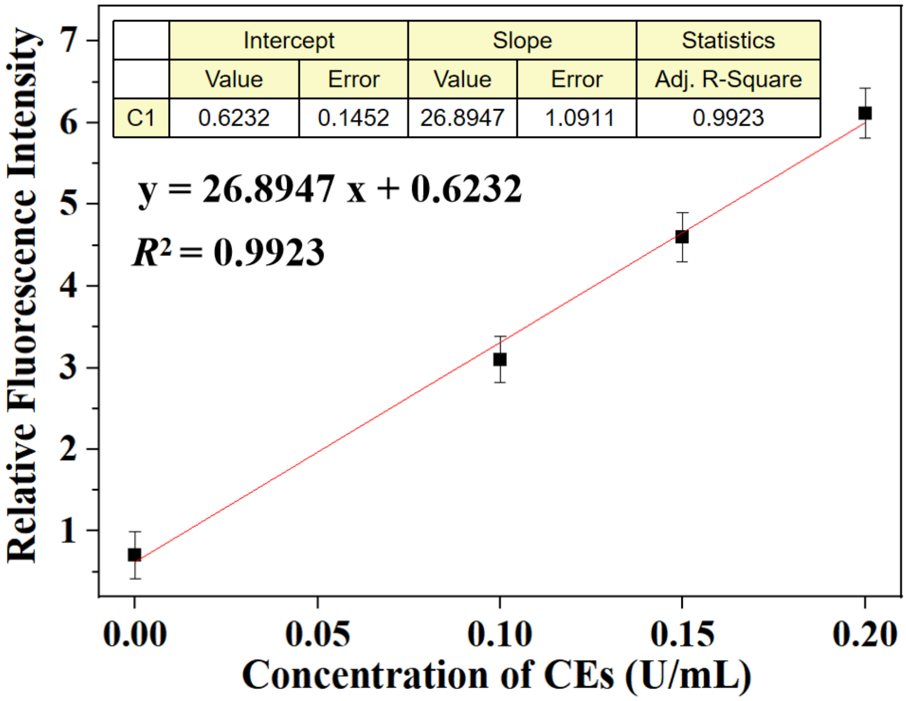

Figure S5. The linear relation between the relative fluorescence intensity of HepG2 cells with **TTAP−AB** and the

concentration of CEs. Error bars: standard deviation (SD), experimental times: $n = 3$.

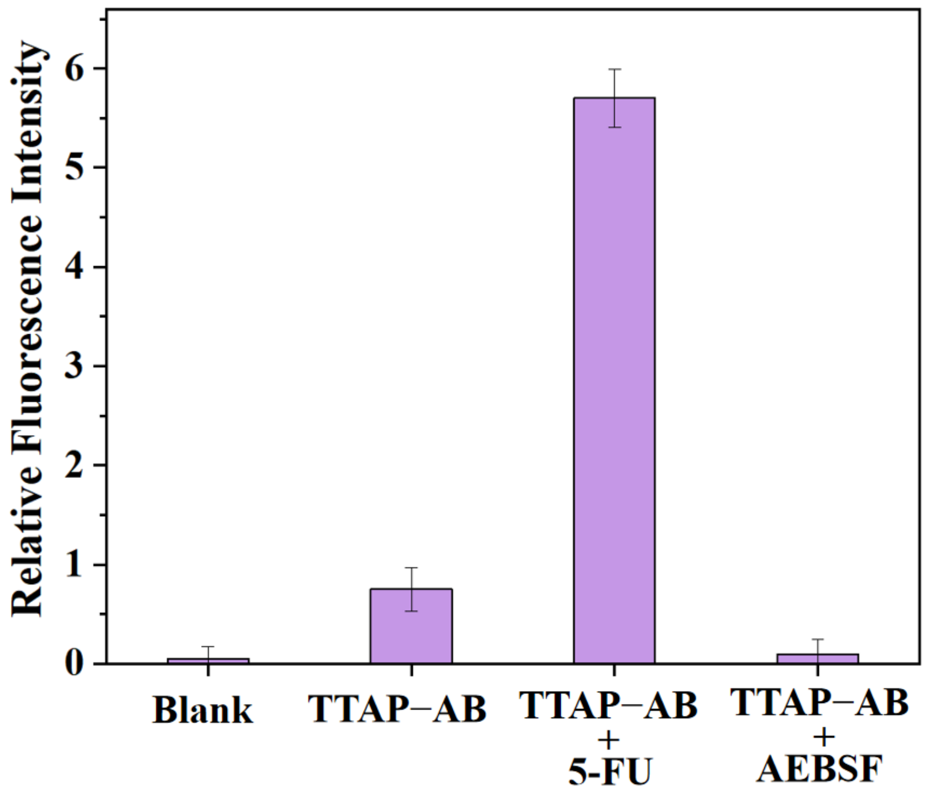

Figure S6. Normalized fluorescence intensity for cell imaging, error bars are ± SD ($n = 3$).

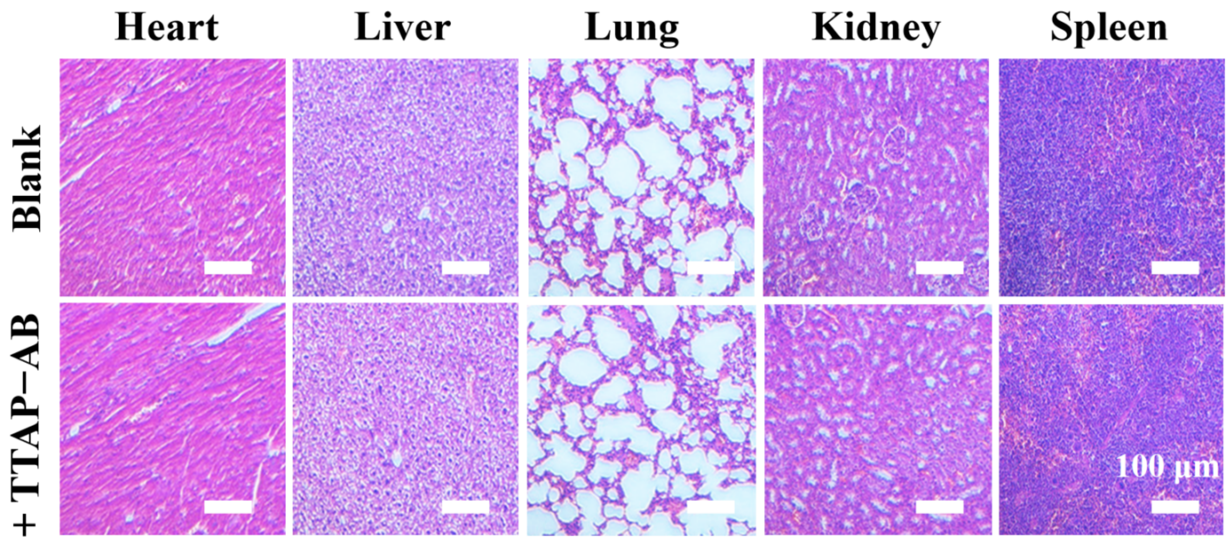

Figure S7. Representative histological sections (H&E staining) for the main organs of mice without and with the injection of the **TTAP−AB** probe (100 μL, 200 μM). Scale bar = 100 μm

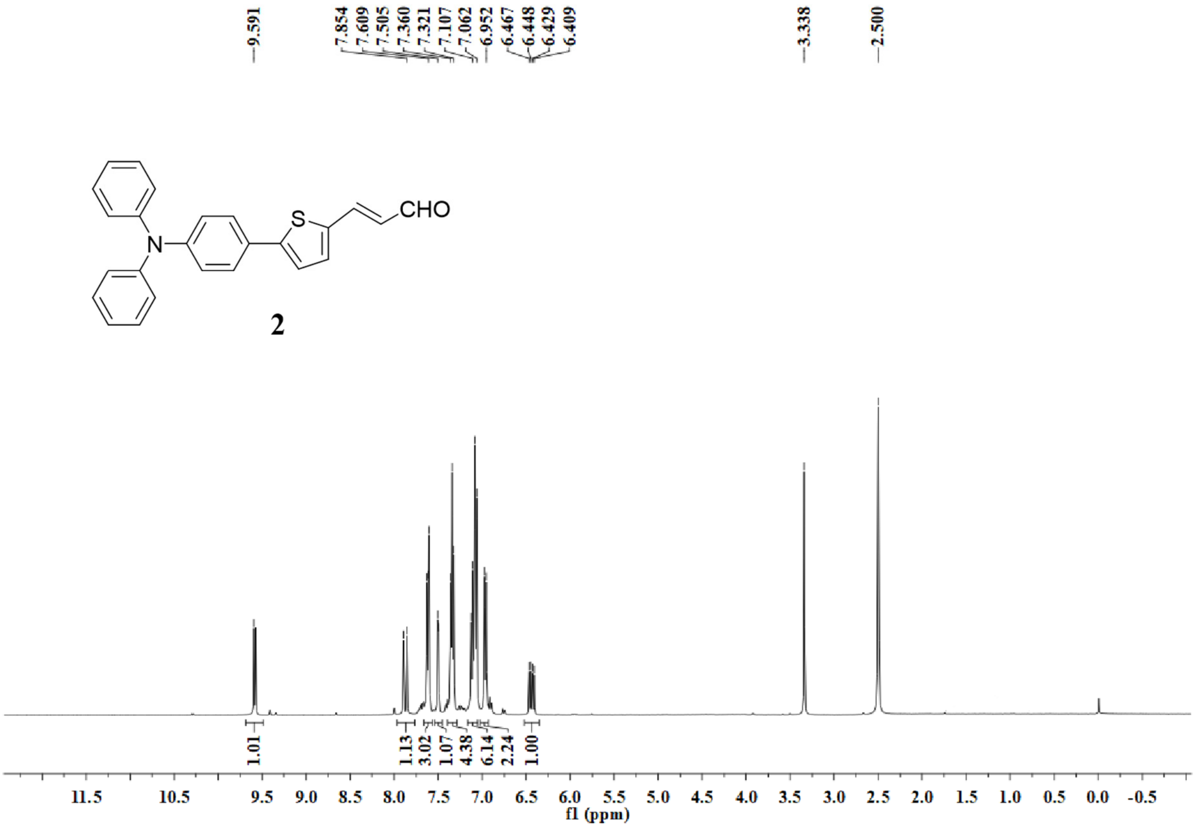

Figure S8. ^{1}H NMR (400 MHz, DMSO-d_6) spectrum of compound **2**

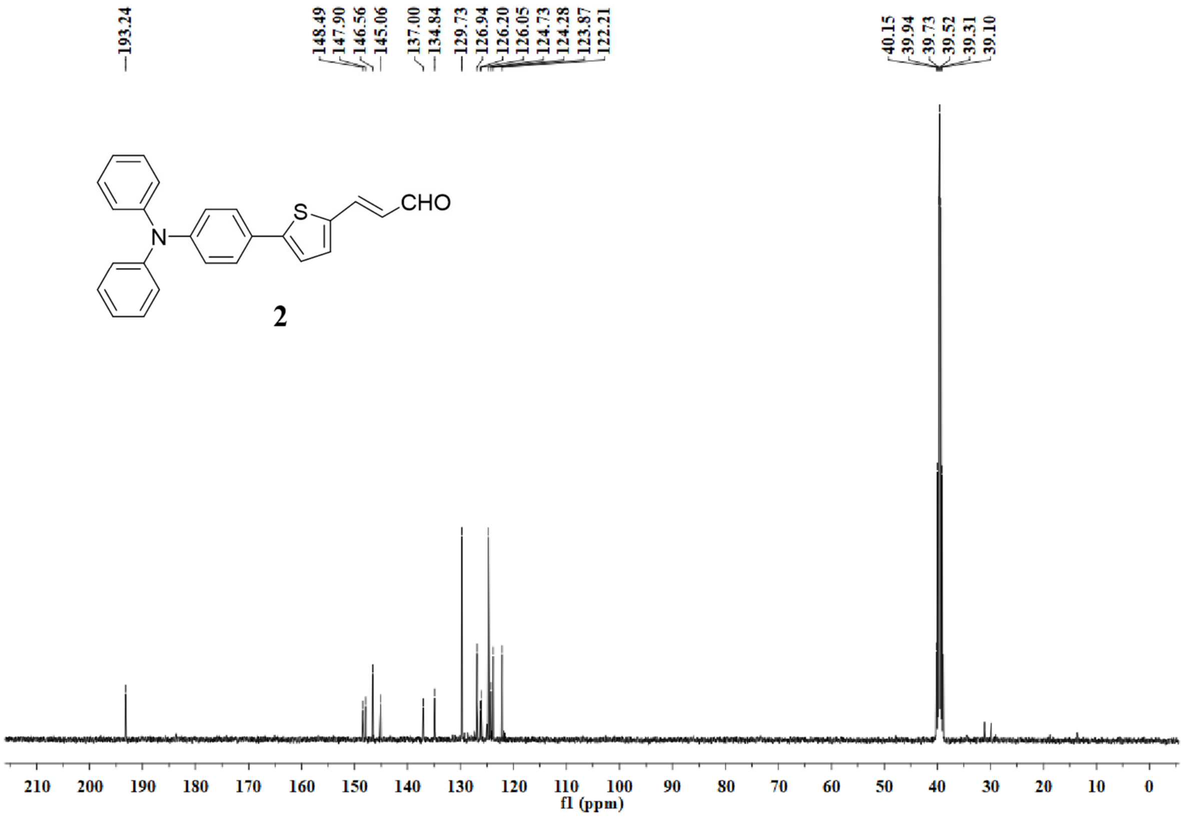

Figure S9. ^{13}C NMR (100 MHz, DMSO-d_6) spectrum of compound **2**

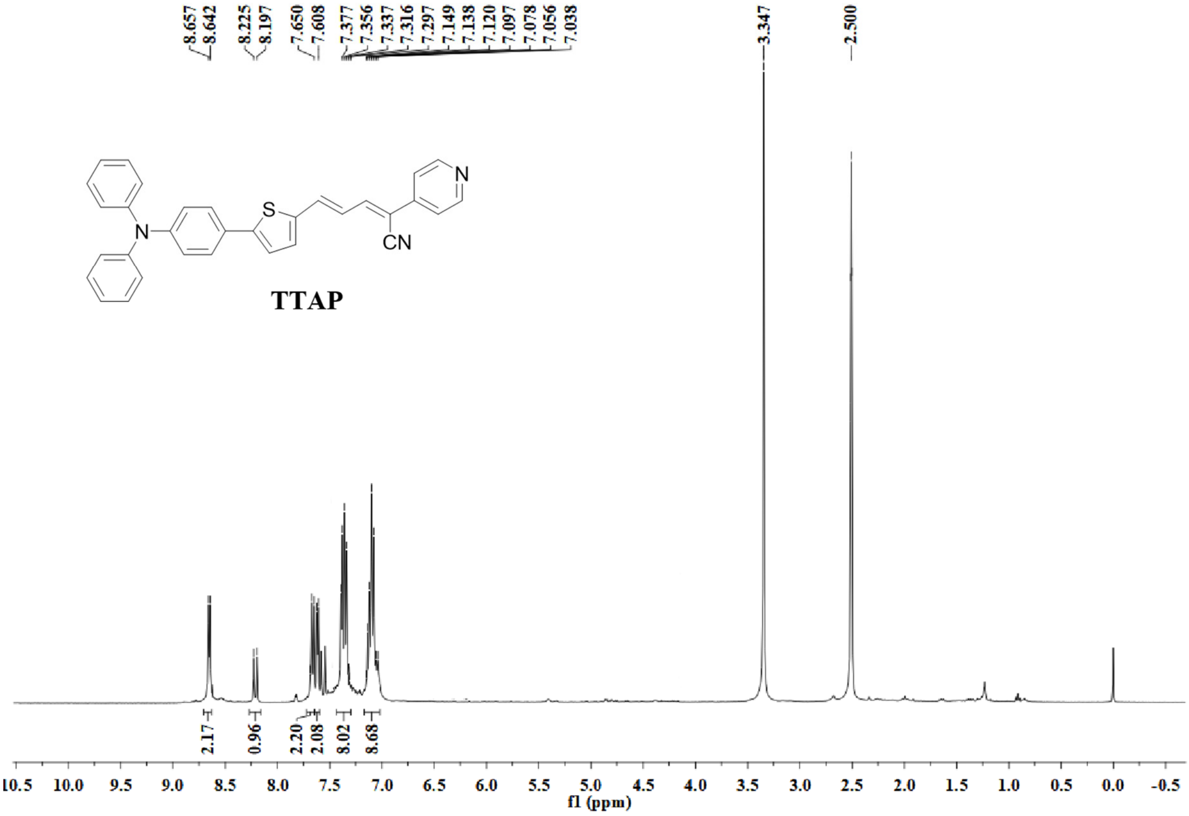

Figure S10. ^{1}H NMR (400 MHz, DMSO-d_6) spectrum of **TTAP**

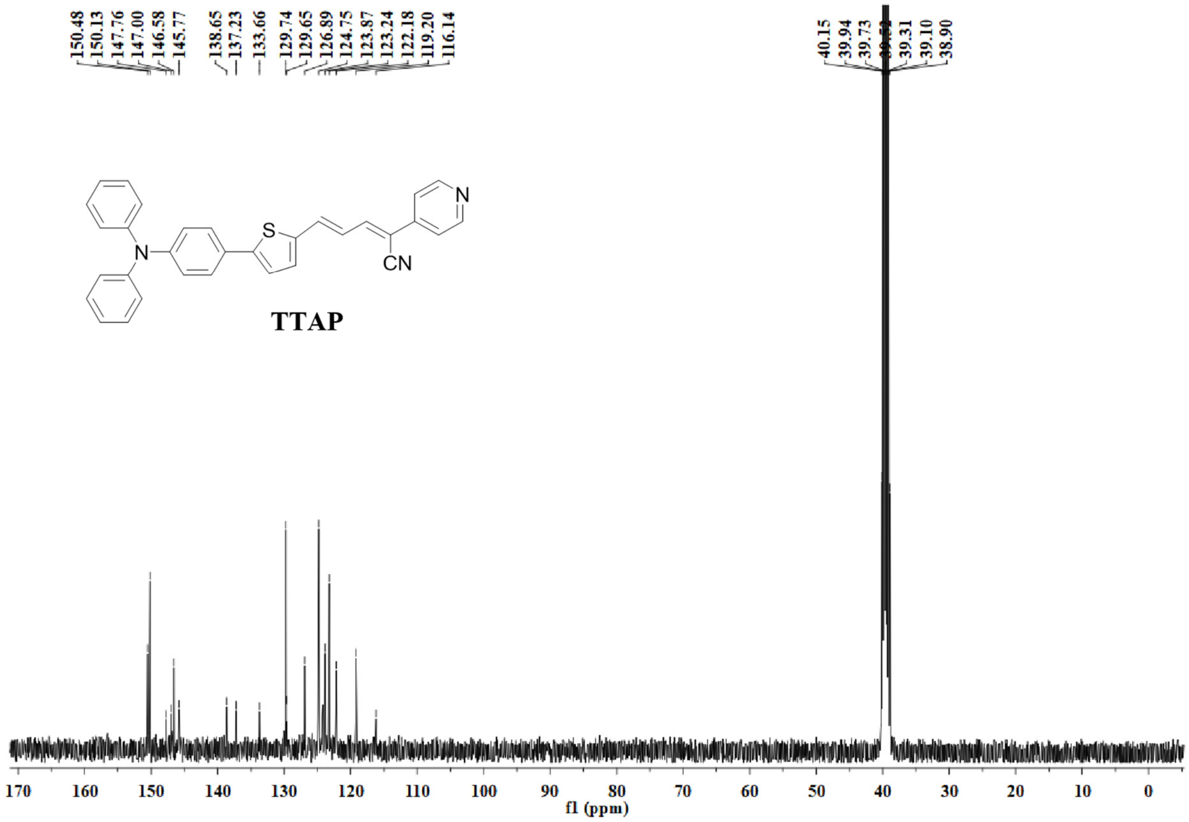

Figure S11. ^{13}C NMR (100 MHz, DMSO-d_6) spectrum of compound **TTAP**

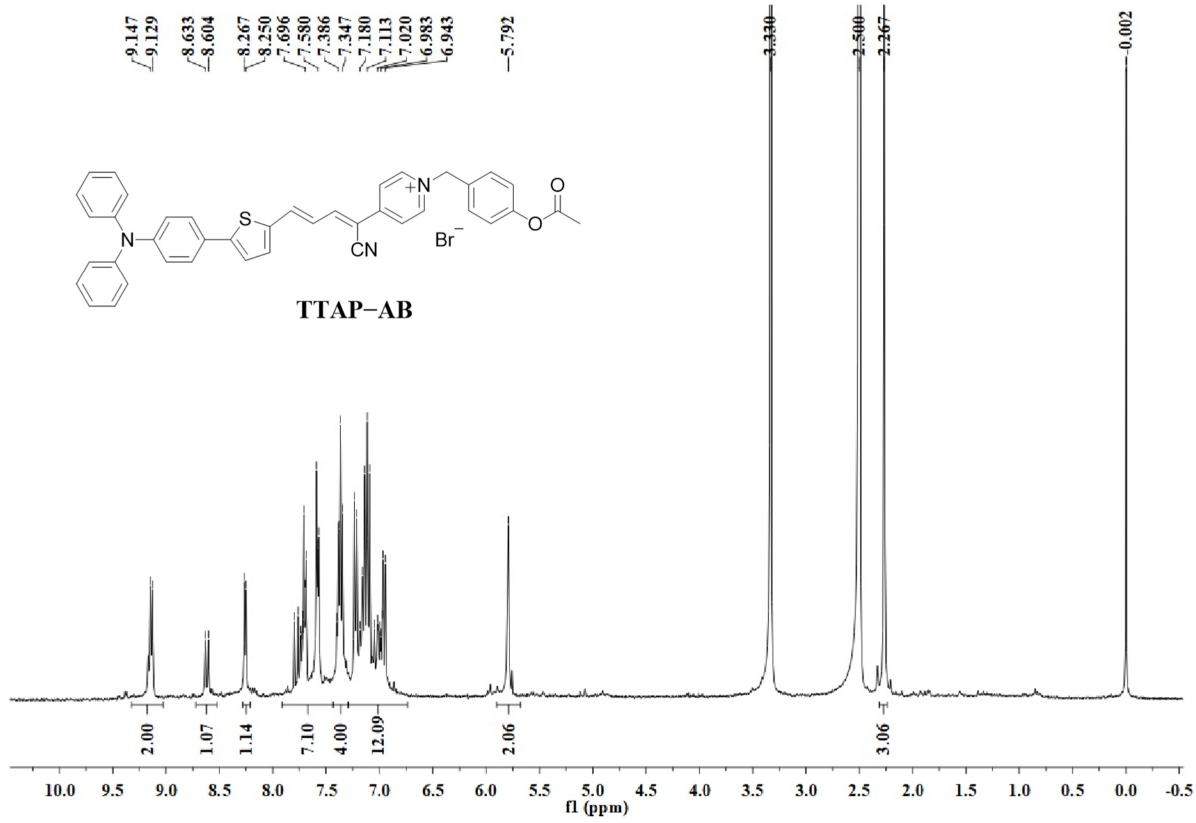

Figure S12. ^{1}H NMR (400 MHz, DMSO-d_6) spectrum of the **TTAP−AB** probe

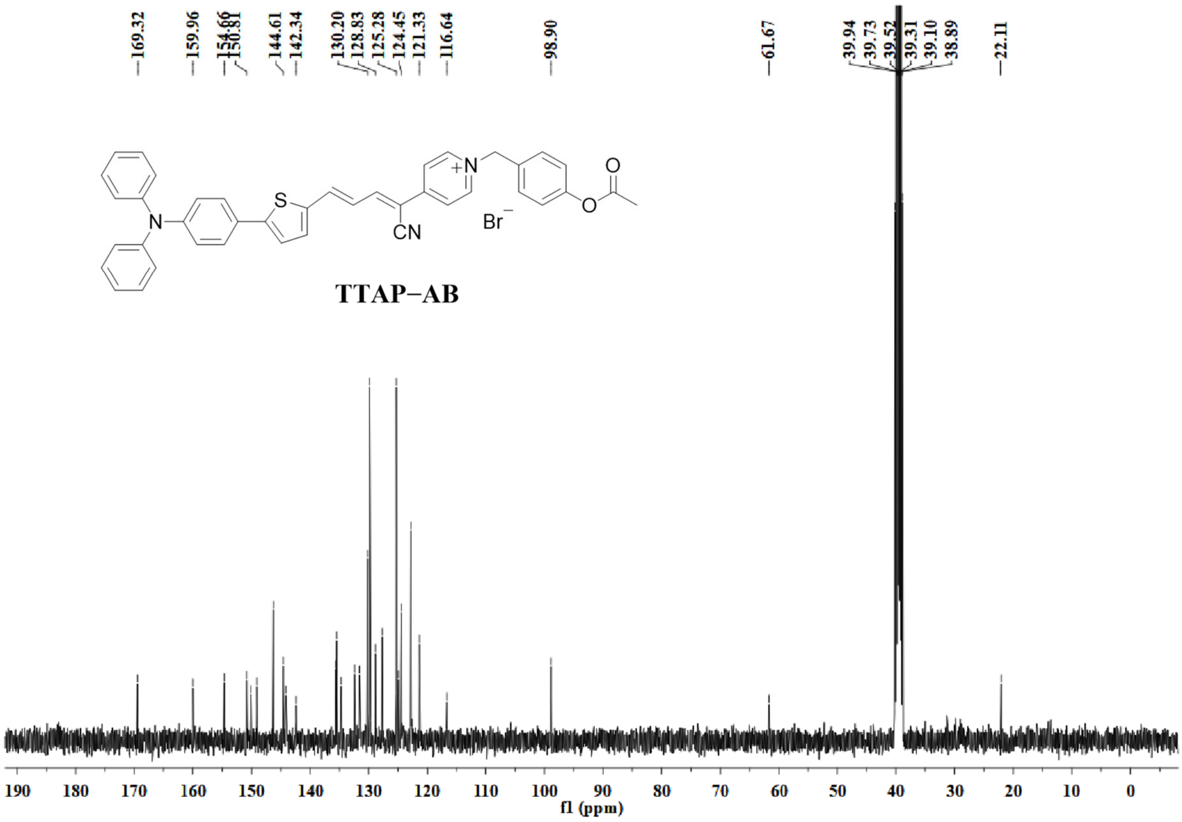

Figure S13. ^{13}C NMR (100 MHz, DMSO-d_6) spectrum of the **TTAP−AB** probe

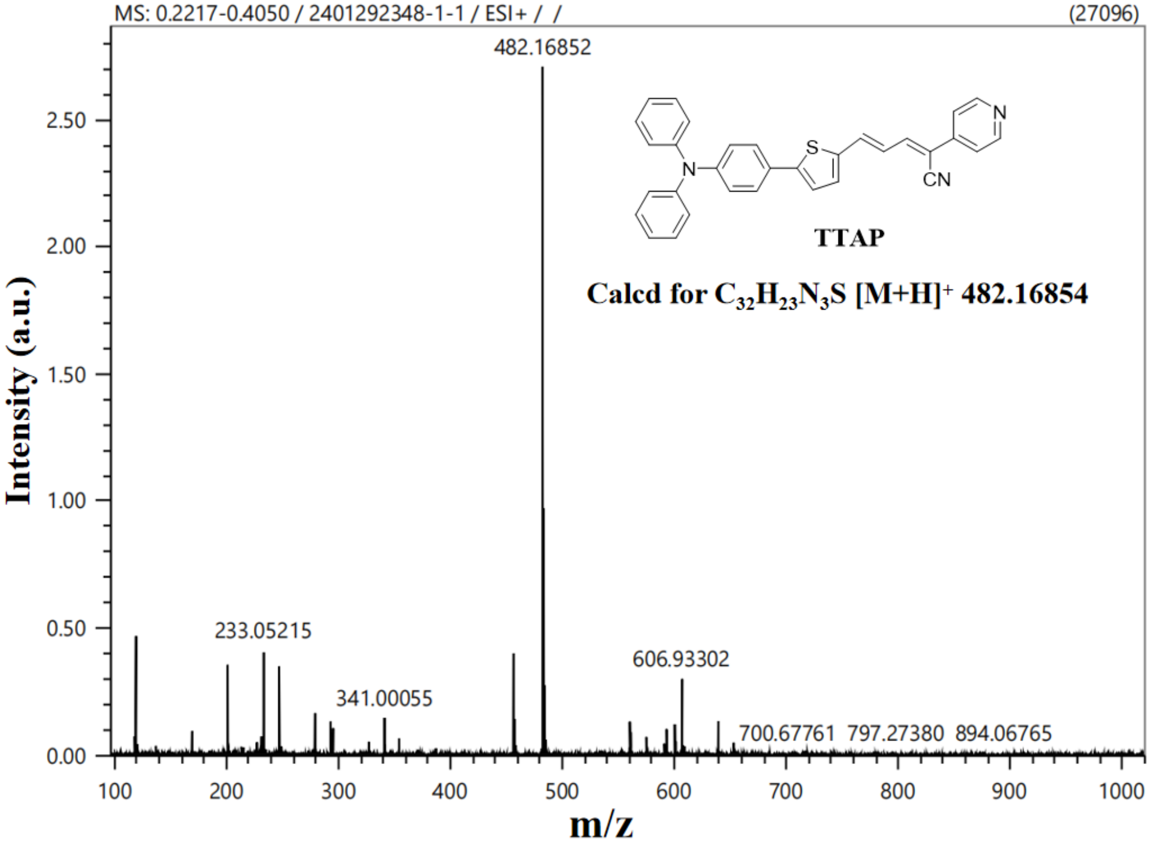

Figure S14. HRMS spectrum of compound **TTAP**

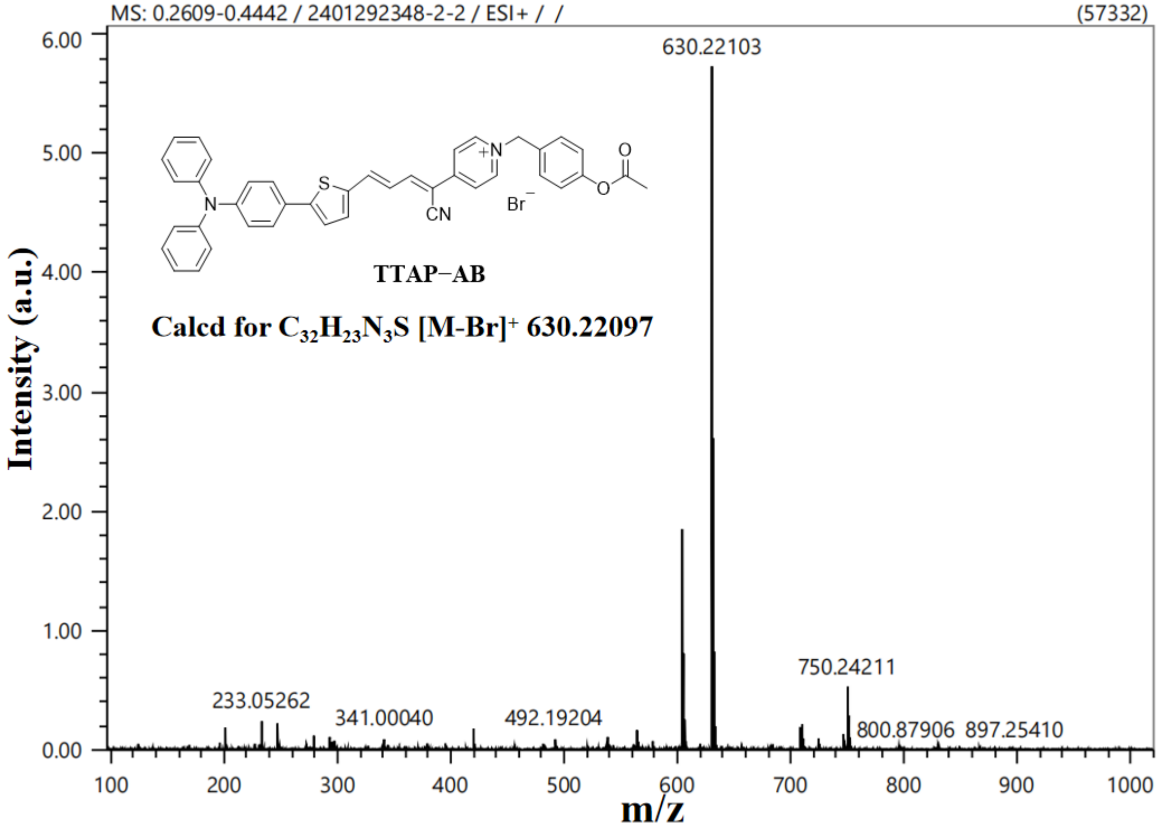

Figure S15. HRMS spectrum of the **TTAP−AB** probe
